# Obstructive Sleep Apnoea and Cardiac Arrhythmias (OSCA) trial: a nested cohort study using injectable loop recorders and Holter monitoring in patients with obstructive sleep apnoea

**DOI:** 10.1136/bmjopen-2022-070884

**Published:** 2023-02-14

**Authors:** Hejie He, Thomas Lachlan, Nakul Chandan, Ven Gee Lim, Peter Kimani, G Andre Ng, Asad Ali, Harpal Randeva, Faizel Osman

**Affiliations:** 1 R&D Institute of Cardio-metabolic Medicine, University Hospitals Coventry and Warwickshire NHS Trust, Coventry, UK; 2 Warwick Medical School, University of Warwick Warwick Medical School, Coventry, UK; 3 University of Warwick Warwick Medical School, Coventry, UK; 4 Warwick Medical School, University of Warwick Faculty of Medicine, Coventry, UK; 5 Cardiovascular Sciences, University of Leicester, Leicester, UK; 6 R&D Institute of Cardiometabolic Medicine, University Hospitals Coventry and Warwickshire NHS Trust, Coventry, UK

**Keywords:** Pacing & electrophysiology, Adult cardiology, Adult thoracic medicine, SLEEP MEDICINE

## Abstract

**Introduction:**

Obstructive sleep apnoea (OSA) is associated with increased cardiovascular mortality despite continuous positive airways pressure (CPAP) therapy. This excess risk may be related to increased arrhythmia risk, especially atrial fibrillation (AF). The true incidence of arrhythmia in patients with OSA is unknown. Implantable loop recorders (ILR) are powerful tools for detecting arrhythmias long-term. Cardiac autonomic function may be important in arrhythmogenesis in these patients but needs further study. We aim to identify the true incidence of arrhythmias (especially AF) using ILRs, assess cardiac autonomic function using Holter monitors in patients with OSA and explore cardiovascular outcomes.

**Methods and analysis:**

A two-centre (University Hospital Coventry and St. Cross Hospital, Rugby) nested cohort study using Reveal LINQ (Medtronic, UK) ILR to identify precise arrhythmia (atrial/ventricular) incidence in patients with moderate–severe OSA. 200 patients will be randomised 1:1 to standard care alone or standard care+ILR (+Holter monitor at baseline and 12 months). The primary objective is to compare arrhythmia detection over 3 years between the two groups. Cardiac autonomic function will be assessed in the ILR-arm at baseline and 12 months post CPAP. Secondary objectives will explore the mechanisms linking OSA and arrhythmia using cardiac autonomic function parameters based on Holter recordings and circulating biomarkers (high sensitivity Troponin-T, N-terminal pro B-type natriuretic peptide, matrix metalloproteinase-9, fibroblast growth factor 23, high sensitivity C-reactive protein, interleukin-6 and tumour necrosis factor-α) before and after CPAP initiation in the ILR-arm.

**Ethics and dissemination:**

This study has been approved by the Health Research Authority after examination by the Solihull Research and Ethics Committee. The main ethical considerations was the minimally invasive nature of ILR insertion outside of usual care. Patient advisory groups were consulted with a positive outcome for this type of research. We plan on publishing papers in peer-reviewed journals based on the primary objective and any interesting findings from secondary objectives. We will endeavour to publish all relevant data.

**Trial registration number:**

NCT03866148.

**Strengths and limitations of this study**

- Obstructive Sleep Apnoea and Cardiac Arrhythmia uses implantable loop recorders (ILR) which are the gold standard in arrhythmia detection thereby determining the incidence of arrhythmias over 3 years in this obstructive sleep apnoea population.
- The combination of cardiac autonomic function surrogates in heart rate variability, heart rate turbulence with biomarkers and arrhythmia burden using the ILR is novel and likely to be hypothesis generating.
- As there are few exclusion criteria, the external validity of this study is likely to be high.
- Due to the nature of nested cohort studies, the causation of arrhythmias cannot be determined.

## Introduction

### Obstructive sleep apnoea and arrhythmias

Obstructive sleep apnoea (OSA) is characterised by abnormal or cessation of breathing during sleep due to narrowing or closure of the upper airways. The estimated prevalence of moderate or severe disease ranges from 5% to 50% with the highest proportion seemingly in overweight middle-aged men.^1–3^ These patients suffer from increased snoring, excessive daytime sleepiness and a higher incidence of cardiovascular disease and arrhythmia, even after controlling for weight and comorbidities (such as diabetes and hypertension) which are prevalent in both OSA and cardiovascular disease. Arrhythmias can compound symptoms of shortness of breath and fatigue in patients with OSA and can lead to stroke, sudden cardiac death (SCD) and heart failure. Increased severity of OSA increases risk of arrhythmia, the most common being atrial fibrillation (AF) with up to 60% of the patients with sleep apnoea having some type of arrhythmia.^4 5^ Some studies of patients with OSA suggest AF in 4.8%^6^ yet one study using an implantable loop recorder (ILR) demonstrated AF in 20% (up to five times higher than the general population).^7^ Other arrhythmias including heart block, bradycardias and ventricular tachycardia (VT) also appear to be more prevalent. Despite this, European guidelines do not specifically encourage screening for sleep apnoea in AF.^8^ Similarly, AF and other arrhythmias are not routinely screened for in patients with OSA.^9^

Continuous positive airways pressure (CPAP) is the mainstay of hospital prescribed treatment. This gives airways support to a patient overnight via a mask or nasal prongs and is proven to improve patient symptoms and the number of hypopnoea and apnoeic episodes.^10^ Its effect on associated morbidity and mortality suggest it reduces episodes of arrhythmia and major cardiovascular events (MACE (defined as death, myocardial infarction, coronary revascularisation, stroke and heart failure)). However, a recent meta-analysis and the SAVE trial involving 2687 patients in the primary analysis demonstrated no significant reduction in cardiovascular outcomes or reduction in AF.^11 12^ Further study is needed to determine what factors prevent CPAP from reducing this morbidity and mortality.^13^

Arrhythmias, most commonly AF, are detected by ECGs or ambulatory ECG monitoring (eg, Holter monitoring). However, these diagnostic tools can often miss significant paroxysmal arrhythmias. Short paroxysms of arrhythmia not detected by standard screening could account for a proportion of the increased morbidity and mortality despite CPAP therapy seen in previous studies. While shown to be effective at near eliminating apnoeas and hypopnoeas, CPAP therapy can be heterogenous in its usage with mean duration of treatment and mask types varying between studies. Underusage of CPAP or differences in therapy types (eg, nasal CPAP vs full-mask CPAP) and therefore incomplete treatment may also contribute. Other less studied mechanisms that link OSA with MACE and arrhythmia could also explain this disparity but need further study.

### Current guidelines

OSA is an independent risk factor for arrhythmia though there is no clear consensus on the overall incidence and mechanisms for this. Current guidelines do not encourage screening for arrhythmia in OSA and vice versa though it is often considered in general workups for patients and their comorbidities though the 2020 European Society of Cardiology AF guidelines does recommend optimisation of OSA for AF management.^8^

### Mechanisms

OSA causes episodic hypoxia and increased intrathoracic pressures which via inflammation, oxidative stress and dysregulation of the autonomic nervous system, associate it with arrhythmia, hypertension, MACE, pulmonary hypertension and SCD.^14 15^ The cardiac autonomic nervous system is a key way the body maintains homoeostasis of the heart and lungs. The sympathetic nervous system ‘fight and flight’ response and the parasympathetic nervous system ‘rest and digest’ responses are antagonistic and imbalances or hyperactivity in both are linked to tachyarrhythmias, particularly AF and VT.^16^ Abnormalities in breathing, especially apnoeic episodes, significantly elevated sympathetic activity and parasympathetic activity.^17^ Pauses in breathing trigger parasympathetic activity via baroreceptors in the lung and cause paroxysmal bradycardias. This can reduce the atrial effective refractory period and promote increased activity in atrial myocytes in the pulmonary vein ostia, leading to AF. Studies in patients with sleep apnoea show that their sympathetic activation is higher throughout the day and not just during sleep.^18^ Increased sympathetic tone can also induce ectopy and increased firing in atrial tissue in the pulmonary veins, again, leading to AF. Parasympathetic and sympathetic activity can be measured using spectral analysis of heart rate variability (HRV) from 24-hour Holter recordings. Cycles of HRV are observed and grouped into ultra-low, very-low, low and high frequency bands. Loss of variability indicates higher risk of cardiovascular disease and arrhythmia. Analysis and comparison of HRV before CPAP initiation and a time after treatment will demonstrate any significant changes to cardiac autonomic control over this time. This can be correlated to arrhythmias identified on ILR. Heart rate turbulence (HRT) will also be derived from Holter data and analysed in the same way as HRV.

Mechanisms linking sleep apnoea and MACE include oxidative stress, endothelial dysfunction, mechanical stress and inflammation during hypoxic episodes in sleep apnoea.^19^ The most researched markers associated with sleep apnoea were high sensitivity C-reactive protein (hs-CRP), tumour necrosis factor-α (TNF-α)^20^ and interleukin-6 (IL-6)^21^ identifying higher levels of inflammation in the body. A recent meta-analysis shows C-reactive protein, TNF-α and IL-6 reduce with CPAP treatment in patients with moderate-to-severe sleep apnoea.^22^ However, all the studies involving TNF-α and IL-6 cited had low numbers of patients and further study with larger numbers is warranted. Other studies revealed matrix metalloproteinase-9 (MMP-9) is a useful marker in identifying hypertension in patients with sleep apnoea. It is associated with oxidative stress and therefore cardiovascular damage and has been shown to be increased in hypoxia.^23^ Increased levels of MMP-9 have been correlated with increasing severity of sleep apnoea. High sensitivity Troponin-T (hsTnT) is a heart specific enzyme which is released on damage to the heart. N-terminal pro B-type natriuretic peptide (NTpro-BNP) is a precursor to brain natriuretic peptide and is released from the walls of the heart in increased tension. Both are useful diagnostic and risk stratifying markers for ischaemia and heart failure, respectively, though there may be some overlap. HsTnT is also linked with increased incidence of AF. In a recent study fibroblast growth factor 23 and NTpro-BNP has been shown to correlate with the development of AF in the general population.^24^ This marker may be an important link between sleep apnoea and AF. A better understanding of the arrhythmia profile, CPAP usage, autonomic dysfunction profile and biomarker profile may help identify what drives the cardiovascular mortality and morbidity in patients with sleep apnoea and help target treatment.

### Continuous remote monitoring and other monitoring

ILRs are small devices used to detect arrhythmias. Once implanted they continuously monitor the heart rhythm and record abnormalities for a period of 3 years or more. The data stored on the device is relayed via Bluetooth to a cellular-connected base-station or cellular phone which then sends the information to the database. Unlike Holter monitoring, they provide a complete extended temporal profile. CPAP usage is also recorded remotely allowing detailed subanalysis of this cohort of patients to determine the effect of treatment. Additionally, measurement of cardiac autonomic function using Holter monitors at baseline and after 12 months of treatment gives another longitudinal view of the processes that could trigger arrhythmias in this group of patients. Identifying arrhythmia incidence in this group using an ILR device has only been performed in a small cohort of 20 patients^7^ (specifically AF in this case) using the Medtronic Reveal XT Device. We plan on using the Medtronic Reveal LINQ which is smaller and less invasive and may improve participation and achieve greater recruitment. This, alongside the biomarkers we are analysing at the same time points may elucidate the disparity between treated CPAP and continuing risk of heart disease and arrhythmia.

## Methods and analysis

The Obstructive Sleep Apnoea and Cardiac Arrhythmia (OSCA) trial is a multihospital randomised nested cohort study. Participants are being recruited from sleep clinic after diagnosis of OSA and prior to CPAP initiation. All participants continue their usual treatment pathway for OSA alongside the trial protocol. A randomisation process occurs at consent, with a 1:1 split with one group receiving an ILR, 24-hour Holter, echocardiogram and blood tests and the other receiving remote follow-up only; this follow-up is repeated in both groups at 12 months and 3 years remotely. The burden of arrhythmia in each group will be compared, with a focus on AF as defined by international guidelines.^8^ Identification of arrhythmia and treatment is implemented by the principal investigators and guided by the Trial Steering Committee (TSC). The arrhythmia profile of those in the ILR group will be correlated to cardiac autonomic function parameters (both HRV and HRT), CPAP usage, echocardiogram findings and vascular biomarker profiles. MACE outcomes will be compared between the groups.

The OSCA trial started recruiting in October 2019 with an 18-month recruitment period and follow-up of 3 years. However, recruitment was halted very soon after due to the COVID-19 pandemic and has only recently restarted. We had recruited 31 participants (16 ILR no ILR) prior to the study being halted.

### Objectives

The primary objectives are to characterise the arrhythmia incidence/burden in an OSA population using ILRs compared with standard care alone (the primary endpoint being clinically significant arrhythmias (of which we would treat)) and to assess cardiac autonomic function at baseline and following 12 months of CPAP therapy in the ILR group.

The secondary objectives are to: (a) compare morbidity (with focus on MACE outcomes) of patients with OSA in both groups, (b) explore frequency and onset of arrhythmias in the ILR-group versus no ILR-group with respect to CPAP usage profile, (c) identify predictors for arrhythmia from all data gathered (including demographics, medical history and measured parameters as above), (d) characterise general cardiovascular and inflammatory biomarkers at initiation and during CPAP in the ILR group and (e) explore patients with OSA in terms of patient reported outcome measures in the ILR group.

### Participants

The study population are those who have been diagnosed with moderate–severe OSA and are referred to start CPAP therapy. CPAP initiation clinics and trial procedures will be conducted at University Hospital Coventry & Warwickshire, UK, and Hospital of St. Cross, Rugby, UK.

### Inclusion and exclusion criteria

#### Inclusions

Patients with moderate-to-severe OSA defined as an Apnoea Hypoponea Index of 15 or above, who require CPAP as standard care and are between the age of 18–75 years.

#### Exclusions

Patients who already have a diagnosis of AF/atrial flutter and/or VT, those with an ILR already in-situ or an established indication for an ILR and those with a palliative diagnosis, that is, life expectancy less than 3 years will be excluded from the trial. Patients who lack capacity cannot consent directly and are therefore excluded.

### Participant pathway flow chart

Figure 1.

**Figure 1:**
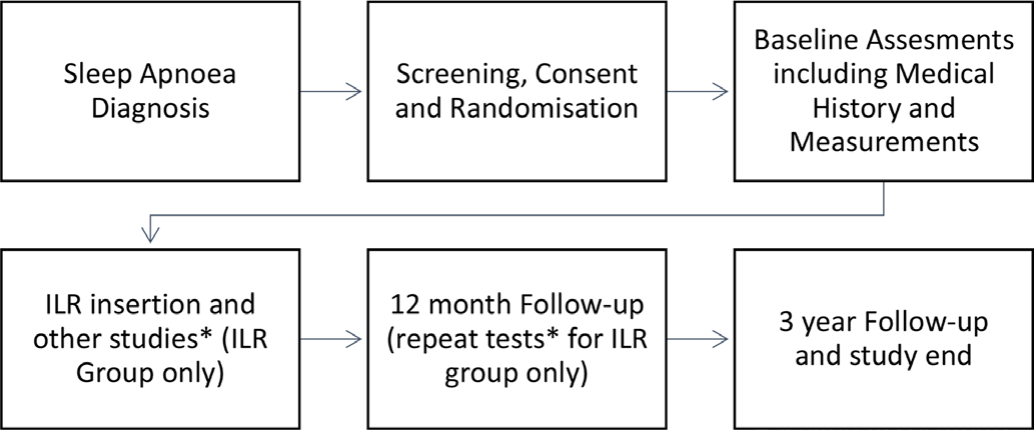
Patientflow diagram. *Holter monitor, blood tests and questionnaires. ILR, implantable loop recorder.

### Screening

Patients who have been newly diagnosed with OSA as an outpatient are identified from the sleep clinic and approached with an information leaflet. If interested, their suitability is assessed in person at the clinic and they are enrolled into the study.

### Eligibility and baseline assessment

Once initial consent is taken, baseline demographics, medical history and medication history is completed and a 12-lead ECG is performed. Eligibility criteria are then checked and confirmed. Randomisation occurs at this point. If the participant is recruited into the ILR arm they have their ILR insertion, blood tests and questionnaires done. Those randomised to standard care alone will not need any further tests. Treatment pathways for OSA remain unchanged for both groups with their regular CPAP troubleshooting sessions continue (usually 1 month after initiation).

### ILR-arm

The ILR insertion is performed by a trained practitioner in the left upper chest wall as per manufacturer guidelines, in this case Reveal LINQ (Medtronic, Minneapolis, USA). Observations and physical measurements are recorded and blood tests taken. Data from the ILR is uploaded on a 24-hour schedule so any clinically significant arrhythmias will be identified and treated promptly as per European Cardiac Society (ESC) guidelines. A 24-hour Holter monitor is applied between initial enrolment and ILR insertion. Data from the 24-hour Holter will then be analysed for arrhythmia, HRV and HRT.

### 12-month follow-up

Participants in the ILR arm are contacted at 12 months with a 2-week window for repeat observations, physical measurements, blood tests and 24-hour Holter to be performed. Initial comparisons of 24 hours Holter data, CPAP usage and arrhythmia detection by ILR can then be performed for each participant. Those in the standard care alone arm are contacted via telephone and a medical history is retaken to identify any incidence of arrhythmia. Their hospital record is then reviewed for any arrhythmia and CPAP usage since enrolment.

The TSC will review any endpoints which are met and inform whether any interim analysis may be required.

### 3-year check and study end

At 3 years all participants are contacted via telephone to check their medical history. Any arrhythmias are documented. Those in the ILR arm are offered ILR removal. Participants are informed of the study end and any interim results will be offered to them. The planned data collection is summarised in table 1.

**Table 1:** Schedule of events

Procedures	Consultant sleep clinic/sleep study	Baseline CPAP clinic	ECHO/24-hour tape*	ILR if not done yet	12 months appointment*	2 years	3 years
Screening	X						
Patient information sheet	X						
Eligibility assessment	X						
Informed consent		X					
Randomisation		X					
Medical history		X†			X*	X	X
Clinical examination		X†					
Bedside observations		X†			X*		
12-lead ECG‡		X			X*		
Health questionnaires (EQ5D)‡		X			X*		
Blood samples (routine, NTpro-BNP, hsTnT, hsCRP IL-6, TNF-α, MMP-9 and FGF-23)‡		X			X*		
Echocardiography—(LV/RV dimensions and functions and other ECHO parameters)§			X*		X*		
24-hour Holter§			X*				
Standard therapy (lifestyle advice, referral to bariatric or weight loss services as usual)		X†					
Continuous positive airway pressure check		X†					
Insertion of injectable loop recorder (ILR)‡		X		X			
ILR remote monitoring‡		X					X
Adverse events (include SAEs—hospital admissions)		X					X
Hospital readmissions and mortality		X					X
Arrhythmia detection and referral for treatment‡		X					X
Removal of ILR							X

*Research activities will be optional and conducted in line with trust COVID-19 safety policy subject to capacity and capability.

†Standard treatment pathway.

‡For ILR group only.

§For ILR group where available.

CPAP, continuous positive airways pressure; EQ5D, European Quality of Life- 5 Dimension; FGF-23, fibroblast growth factor-23; hsCRP, high-sensitivity C-reactive protein; hsTnT, high sensitivity Troponin-T; LV, left ventricle; MMP-9, matrix metalloproteinase-9; NTpro-BNP, N-terminal pro B-type natriuretic peptide; RV, right ventricle; SAE, serious adverse event; TNF-α, tumour necrosis factor-α.

#### Patient and public involvement

Feedback and design input was sought from our local public and patient involvement groups on two occasions. The first was feedback on a preliminary patient information sheet (PIS) detailing the study. These perspectives changed the way the PIS was formatted and worded. It also streamlined the patient pathway to reduce the number of hospital visits.

The second session concluded with an agreement that a general patient would be happy to take part in the study as it would have additional monitoring benefits as well as screening them for other medical issues. They asked about how the research team would minimise extra patient travel and whether participants would receive any reimbursement for travel outside standard care. The Reveal LINQ device safety was discussed, and the group felt that other than initial insertion it should not be an issue for participants.

A member of this group was asked to join the TSC.

### Power calculation

The trial will recruit 200 participants based on AF incidence of 20%^7^ using an ILR and 5% using traditional AF detection methods. This gives a 0.99 power with an alpha of 0.05 when comparing incidence of arrhythmia in the ILR arm compared with the standard care alone arm.

### Statistical analysis

For the primary objective of comparing arrhythmias detection rates between ILR group and standard care group, a χ^2^ test or a Fisher’s test will be used. For the co-primary objective of comparing mean HRT/HRV at baseline and 12 months in the ILR group, a paired t-test will be used. Mean difference and 95% CI will also be reported.

For secondary objectives: (a) Compare MACE between both arms at follow-up using a χ^2^ test or a Fisher’s exact test as appropriate. (b) Correlate arrhythmias detected in ILR at follow-up with effective CPAP usage using a χ^2^ test or a Fisher’s exact test as appropriate. (c) For the ILR group, we will identify predictors for arrhythmia. To identify predictors and with outcome being whether a patient has arrhythmia during the study (yes/no), we will fit simple and multiple logistic regression models. The explanatory variables in the logistic models will be the potential predictors for arrhythmia collected at baseline visit including age, gender, comorbidity, alcohol/smoking history, HRV and HRT parameters. (d) Compare vascular biomarkers before and after CPAP in the ILR arm using paired t-tests. (e) Perform longitudinal analysis of patient reported outcome measures. All tests will be performed at 5% significance level.

### Ethics and dissemination

#### Ethics

The Health Research Authority and Solihull Research Ethics Committee (18//WM/0129) have approved the trial design and research protocol. All participants will have informed consent obtained in writing by an investigator. The main ethical considerations are to do with the minimally invasive nature of ILR insertion which would be beyond standard care for patients with OSA. This was discussed in detail at a patient and participant research advisory group meeting who felt it was appropriate for the type of research. It was noted that it was a short, minimally-invasive procedure and could potentially identify a heart rhythm problem early which would benefit the participant. Other considerations were additional blood tests and non-invasive monitoring which were deemed not to be a significant additional burden to participants due to only two additional visits beyond standard care.

#### Monitoring

The trial is being conducted in accordance with Good Clinical Practice, UK Law and the Declaration of Helsinki 2002. The TSC consists of sleep, respiratory and arrhythmia specialists and a member from the patients and public engagement committee. This group will meet annually to evaluate progress of the study in terms of recruitment, endpoints and adverse events. Publication of results will be overseen by the TSC. Protocol amendments will be submitted by the investigators with the sponsor to Research Ethics Committee as required.

#### Dissemination and data statement

The full data set, study protocol and consent material will be anonymised and published in a suitable open data bank. We will aim to publish the primary objective of arrhythmia incidence as detected by ILR in a peer-reviewed journal with copies distributed to participants in the study and to the patient advisory group. Any findings relating to each individual will be shared with the relevant participant. We aim to publish details of arrhythmia burden related to cardiac autonomic function and CPAP usage in peer-reviewed journals.

## Supplementary Material

Reviewer comments

Author's manuscript

## References

[1] Davies RJ, Stradling JR. The epidemiology sleep apnoea introductory article. Thorax 1996;51:65–70. 10.1136/thx.51.Suppl_2.S65 PMC1090710 8869356

[2] Punjabi NM. The epidemiology of adult obstructive sleep apnea. Proc Am Thorac Soc 2008;5:136–43. 10.1513/pats.200709-155MG 18250205 PMC2645248

[3] Benjafield AV, Ayas NT, Eastwood PR, et al. Estimation of the global prevalence and burden of obstructive sleep apnoea: a literature-based analysis. Lancet Respir Med 2019;7:687–98. 10.1016/S2213-2600(19)30198-5 31300334 PMC7007763

[4] Selim BJ, Koo BB, Qin L, et al. The association between nocturnal cardiac arrhythmias and sleep-disordered breathing: the DREAM study. J Clin Sleep Med 2016;12:829–37. 10.5664/jcsm.5880 26951420 PMC4877315

[5] Bayram NA, Çi̇ftçi̇ B, Güven SF, et al. Prevalence of cardiac arrhythmia in obstructive sleep apnea syndrome. Turk J Med Sci 2010;40:843–50. 10.3906/sag-0910-355

[6] Mehra R, Benjamin EJ, Shahar E, et al. Association of nocturnal arrhythmias with sleep-disordered breathing: the sleep heart health study. Am J Respir Crit Care Med 2006;173:910–6. 10.1164/rccm.200509-1442OC 16424443 PMC2662909

[7] Yeung C, Drew D, Hammond S, et al. Extended cardiac monitoring in patients with severe sleep apnea and NO history of atrial fibrillation (the reveal XT-SA study). Am J Cardiol 2018;122:1885–9. 10.1016/j.amjcard.2018.08.032 30274768

[8] Hindricks G, Potpara T, Dagres N, et al. 2020 ESC guidelines for the diagnosis and management of atrial fibrillation developed in collaboration with the European association of cardio-thoracic surgery (EACTS) the task force for the diagnosis and management of atrial fibrillation of the Europea. Eur Heart J 2021;42:373–498. 10.1093/eurheartj/ehaa612 32860505

[9] Marulanda-Londoño E, Chaturvedi S. The interplay between obstructive sleep apnea and atrial fibrillation. Front Neurol 2017;8:668. 10.3389/fneur.2017.00668 29312113 PMC5732262

[10] Patil SP, Ayappa IA, Caples SM, et al. Treatment of adult obstructive sleep apnea with positive airway pressure: an American Academy of sleep medicine systematic review, meta-analysis, and grade assessment. J Clin Sleep Med 2019;15:301–34. 10.5664/jcsm.7638 30736888 PMC6374080

[11] McEvoy RD, Antic NA, Heeley E, et al. CPAP for prevention of cardiovascular events in obstructive sleep apnea. N Engl J Med 2016;375:919–31. 10.1056/NEJMoa1606599 27571048

[12] Yu J, Zhou Z, McEvoy RD, et al. Association of positive airway pressure with cardiovascular events and death in adults with sleep apnea: a systematic review and meta-analysis. JAMA 2017;318:156–66. 10.1001/jama.2017.7967 28697252 PMC5541330

[13] Drager LF, McEvoy RD, Barbe F, et al. Sleep apnea and cardiovascular disease: lessons from recent trials and need for team science. Circulation 2017;136:1840–50. 10.1161/CIRCULATIONAHA.117.029400 29109195 PMC5689452

[14] Bradley TD, Floras JS. Obstructive sleep apnoea and its cardiovascular consequences. Lancet 2009;373:82–93. 10.1016/S0140-6736(08)61622-0 19101028

[15] Somers VK, White DP, Amin R, et al. Sleep apnea and cardiovascular disease: an American heart association/American college of cardiology foundation scientific statement from the American heart association council for high blood pressure research professional education committee, council on clinical cardiology, stroke council, and council on cardiovascular nursing. In collaboration with the national heart, lung, and blood institute national center on sleep disorders research (National institutes of health). Circulation 2008;118:1080–111. 10.1161/CIRCULATIONAHA.107.189375 18725495

[16] Shen MJ, Zipes DP. Role of the autonomic nervous system in modulating cardiac arrhythmias this review is in a thematic series on the autonomic nervous system and the cardiovascular system, which includes the following articles: role of the autonomic nervous system in modula. Circ Res 2014;114:1004–21. 10.1161/CIRCRESAHA.113.302549 24625726

[17] Khoo MCK, Blasi A. Sleep-related changes in autonomic control in obstructive sleep apnea: a model-based perspective. Respir Physiol Neurobiol 2013;188:267–76. 10.1016/j.resp.2013.05.017 23707878 PMC3778082

[18] Somers VK, Dyken ME, Clary MP, et al. Sympathetic neural mechanisms in obstructive sleep apnea. J Clin Invest 1995;96:1897–904. 10.1172/JCI118235 7560081 PMC185826

[19] Javaheri S, Barbe F, Campos-Rodriguez F, et al. Sleep apnea: types, mechanisms, and clinical cardiovascular consequences. J Am Coll Cardiol 2017;69:841–58. 10.1016/j.jacc.2016.11.069 28209226 PMC5393905

[20] Wang J, Yu W, Gao M, et al. Impact of obstructive sleep apnea syndrome on endothelial function, arterial stiffening, and serum inflammatory markers: an updated meta-analysis and metaregression of 18 studies. J Am Heart Assoc 2015;4:e002454. 10.1161/JAHA.115.002454 26567373 PMC4845236

[21] Imani MM, Sadeghi M, Khazaie H, et al. Evaluation of serum and plasma interleukin-6 levels in obstructive sleep apnea syndrome: a meta-analysis and meta-regression. Front Immunol 2020;11:1343. 10.3389/fimmu.2020.01343 32793188 PMC7385225

[22] Baessler A, Nadeem R, Harvey M, et al. Treatment for sleep apnea by continuous positive airway pressure improves levels of inflammatory markers-a meta-analysis. J Inflamm (Lond) 2013;10:13. 10.1186/1476-9255-10-13 23518041 PMC3637233

[23] Wang S, Li S, Wang B, et al. Matrix metalloproteinase-9 is a predictive factor for systematic hypertension and heart dysfunction in patients with obstructive sleep apnea syndrome. Biomed Res Int 2018;2018:1569701. 10.1155/2018/1569701 29693002 PMC5859852

[24] Navarro-García JA, Delgado C, Fernández-Velasco M, et al. Fibroblast growth factor-23 promotes rhythm alterations and contractile dysfunction in adult ventricular cardiomyocytes. Nephrol Dial Transplant 2019;34:1864–75. 10.1093/ndt/gfy392 30629224

