# Supplementary material for: Obstructive Sleep Apnoea and Cardiac Arrhythmias (OSCA) trial: a nested cohort study using injectable loop recorders and Holter monitoring in patients with obstructive sleep apnoea
Source: BMJ Open. 2023 Feb 14;13(2):e070884. doi: 10.1136/bmjopen-2022-070884 (PMC9950886; doi:10.1136/bmjopen-2022-070884)
Supplement: Author's manuscript [file bmjopen-2022-070884.draft_revisions.pdf]

BMJ Open is committed to open peer review. As part of this commitment we make the peer review history of every article we publish publicly available.

When an article is published we post the peer reviewers' comments and the authors' responses online. We also post the versions of the paper that were used during peer review. These are the versions that the peer review comments apply to.

The versions of the paper that follow are the versions that were submitted during the peer review process. They are not the versions of record or the final published versions. They should not be cited or distributed as the published version of this manuscript.

BMJ Open is an open access journal and the full, final, typeset and author-corrected version of record of the manuscript is available on our site with no access controls, subscription charges or pay-per-view fees (http://bmjopen.bmj.com).

If you have any questions on BMJ Open's open peer review process please email info.bmjopen@bmj.com

BMJ Open

PROTOCOL: Obstructive Sleep Apnoea and Cardiac Arrhythmias (OSCA) Trial – A randomized control study using Injectable Loop Recorders and Holter monitoring in Obstructive Sleep Apnoea Patients

Journal:	*BMJ Open*
Manuscript ID	bmjopen-2022-070884
Article Type:	Protocol
Date Submitted by the Author:	08-Dec-2022

Complete List of Authors:	He, Hejie; University Hospitals Coventry and Warwickshire NHS Trust, Cardiology; University of Warwick Warwick Medical School, Warwick Medical School<br>Lachlan, Thomas; University Hospitals Coventry and Warwickshire NHS Trust, Cardiology; University of Warwick Warwick Medical School,<br>Chandan, Nakul; University Hospitals Coventry and Warwickshire NHS Trust, Cardiology<br>Lim, Ven; University Hospitals Coventry and Warwickshire NHS Trust, Cardiology; University of Warwick Warwick Medical School, Warwick Medical School<br>Kimani, Peter; University of Warwick Faculty of Medicine, Warwick Medical School<br>Ng, G Andre; University of Leicester, Cardiovascular Sciences<br>Ali, Asad; University Hospitals Coventry and Warwickshire NHS Trust, Cardiology<br>Randeva, Harpal; University Hospitals Coventry and Warwickshire NHS Trust, Cardiology; University of Warwick Warwick Medical School, Warwick Medical School<br>Osman, Faizel; University Hospitals Coventry and Warwickshire NHS Trust, Cardiology; University of Warwick Warwick Medical School,

Keywords:	Pacing & electrophysiology < CARDIOLOGY, Adult cardiology < CARDIOLOGY, Adult thoracic medicine < THORACIC MEDICINE, SLEEP MEDICINE

SCHOLARONE™
Manuscripts

PROTOCOL: Obstructive Sleep Apnoea and Cardiac Arrhythmias (OSCA) Trial – A nested cohort study using Injectable Loop Recorders and Holter monitoring in Obstructive Sleep Apnoea Patients

Authors:

Hejie He MRCP[1]
Cardiology Research Fellow

Thomas Lachlan MRCP[1]
Cardiology Research Fellow

Nakul Chandan MRCP[1]
Cardiology Research Fellow

Ven Lim MRCP PhD[1]
Cardiology Clinical lecturer

Peter K Kimani BSc MSc PhD[2]
Associate Professor in Medical Statistics

G Andre Ng PhD, FRCP, FEHRA[3]
Professor of Cardiac Electrophysiology
Consultant Cardiologist / Electrophysiologist

Asad Ali MD FRCP[1]
Consultant Sleep and Respiratory Physician
Lead for Sleep Medicine

Harpal S Randeva FRCP, FAcadTM, PhD[4,5]
Director of R&D, Professor of Medicine
Head & Clinical Lead for Diabetes, Endocrinology & Metabolism

Faizel Osman MD FRCP FESC[1,2]
Consultant Cardiologist /Electrophysiologist
Honorary Professor of Cardiology

Address:

[1]Department of Cardiology
University Hospital Coventry
Clifford Bridge Road
Coventry
CV2 2DX
United Kingdom

[2]Warwick Medical School
University of Warwick
Gibbet Hill Road
Coventry CV4 7HL
United Kingdom

For peer review only

[3]Department of Cardiovascular Sciences
University of Leicester
NIHR Leicester Biomedical Research Centre
Glenfield Hospital Leicester
LE3 9QP

[4]Warwickshire Institute for the Study of Diabetes, Endocrinology and Metabolism (WISDEM), University Hospitals Coventry and Warwickshire NHS Trust, Coventry,
CV2 2DX
United Kingdom

[5]Aston Medical School, College of Health and Life Sciences, Aston University, Birmingham
B4 7ET
United Kingdom

Corresponding author: Professor Faizel Osman
Tel: +44 (0) 2476 965668
Email: faizel.osman@uhcw.nhs.uk
Abstract Word count: 299
Text Word Count: 2943
Conflicts of interest: none

Keywords: CPAP, Obstructive Sleep Apnoea, Arrhythmia, Heart Rate Variability, Atrial Fibrillation, Cardiovascular Biomarkers

Protocol version: 2.1
Protocol Date: 27/01/2021

Funding: British Heart Foundation PG/22/10931
Medtronic ERP-2018-11257

For peer review only

ABSTRACT

Introduction: Obstructive sleep apnoea (OSA) is associated with increased cardiovascular morality despite continuous airways pressure (CPAP) therapy. This excess risk may be related to increased arrhythmia risk, especially atrial fibrillation (AF). The true incidence of arrhythmia in OSA patients is unknown. Implantable loop recorders (ILR) are powerful tools for detecting arrhythmias long-term. Cardiac autonomic function may be important in arrhythmogenesis in these patients but needs further study. We aim to identify the true incidence of arrhythmias (especially AF) using ILRs, assess cardiac autonomic function using Holter monitors in OSA patients and explore cardiovascular outcomes.

Methods and Analysis: A two-centre (University Hospital Coventry and St. Cross Hospital, Rugby) randomised controlled trial using Reveal LINQ (Medtronic, UK) ILR to identify precise arrhythmia (atrial/ventricular) incidence in moderate-severe OSA patients. 200 patients will be randomised 1:1 to standard care alone or standard care + ILR (+Holter monitor at baseline and 12months). The primary objective is to compare arrhythmia detection over 3years between the two groups. Cardiac autonomic function will be assessed in the ILR-arm at baseline and 12-months post CPAP. Secondary objectives will explore the mechanisms linking OSA and arrhythmia using cardiac autonomic function parameters based on Holter recordings and circulating biomarkers (hs-TnT, NT-proBNP, MMP-9, FGF-23, hs-CRP IL-6, TNF-α) before and after CPAP initiation in the ILR-arm.

Ethics and Dissemination: This study has been approved by the Health Research Authority after examination by the Solihull Research and Ethics Committee. The main ethical considerations was the minimally invasive nature of ILR insertion outside of usual care. Patient advisory groups were consulted with a positive outcome for this type of research. We plan on publishing papers in peer-reviewed journals based on the primary objective and any interesting findings from secondary objectives. We will endeavour to publish all relevant data.

Registration: ClinicalTrials.gov (NCT03866148) and EudraCT (2019-002995-15).

For peer review only

Strengths and limitations of this study

- OSCA uses implantable loop recorders which are the gold standard in arrhythmia detection thereby determining the incidence of arrhythmias over 3 years in this obstructive sleep apnoea population.
- The combination of cardiac autonomic function surrogates in heart rate variability, heart rate turbulence with biomarkers and arrhythmia burden using the ILR is novel and likely to be hypothesis generating.
- As there are few exclusion criteria, the external validity of this study is likely to be high.
- Due to the nature of nested cohort studies, the causation of arrhythmias cannot be determined.

For peer review only

INTRODUCTION

Obstructive Sleep Apnoea and Arrhythmias

Obstructive sleep apnoea (OSA) is characterised by abnormal or cessation of breathing during sleep due to narrowing or closure of the upper airways. The estimated prevalence of moderate or severe disease ranges from 5 to 50% with the highest proportion seemingly in overweight middle-aged men.[1–3] These patients suffer from increased snoring, excessive daytime sleepiness and a higher incidence of cardiovascular disease and arrhythmia, even after controlling for weight and comorbidities (such as diabetes and hypertension) which are prevalent in both OSA and cardiovascular disease. Arrhythmias can compound symptoms of shortness of breath and fatigue in OSA patients and can lead to stroke, sudden cardiac death (SCD) and heart failure. Increased severity of OSA increases risk of arrhythmia, the most common being atrial fibrillation (AF) with up to 60% of patients with sleep apnoea having some type of arrhythmia.[4,5] Some studies of OSA patients suggest AF in 4.8%[6] yet one study using an implantable loop recorder (ILR) demonstrated AF in 20% (up to 5 times higher than the general population).[7] Other arrhythmias including heart block, bradycardias and ventricular tachycardia also appear to be more prevalent. Despite this, European guidelines do not specifically encourage screening for sleep apnoea in AF.[8] Similarly, AF and other arrhythmias are not routinely screened for in OSA patients.[9]

Continuous positive airways pressure (CPAP) is the mainstay of hospital prescribed treatment. This gives airways support to a patient overnight via a mask or nasal prongs and is proven to improve patient symptoms and the number of hypopnoea and apnoeic episodes.[10] Its effect on associated morbidity and mortality suggest it reduces episodes of arrhythmia and major cardiovascular events (MACE [defined as death, myocardial infarction, coronary revascularisation, stroke and heart failure]). However, a recent meta-analysis and the SAVE trial involving 2,687 patients in the primary analysis demonstrated no significant reduction in

For peer review only

cardiovascular outcomes or reduction in atrial fibrillation.[11,12] Further study is needed to determine what factors prevent CPAP from reducing this morbidity and mortality.[13]

Arrhythmias, most commonly AF, are detected by ECGs or ambulatory ECG monitoring (e.g. Holter monitoring). However, these diagnostic tools can often miss significant paroxysmal arrhythmias. Short paroxysms of arrhythmia not detected by standard screening could account for a proportion of the increased morbidity and mortality despite CPAP therapy seen in previous studies. Whilst shown to be effective at near eliminating apnoeas and hypopnoeas, CPAP therapy can be heterogenous in its usage with mean duration of treatment and mask types varying between studies. Underusage of CPAP or differences in therapy types (e.g. nasal CPAP vs full-mask CPAP) and therefore incomplete treatment may also contribute. Other less studied mechanisms that link OSA with MACE and arrhythmia could also explain this disparity but need further study.

Current Guidelines

OSA is an independent risk factor for arrhythmia though there is no clear consensus on the overall incidence and mechanisms for this. Current guidelines do not encourage screening for arrhythmia in OSA and vice versa though it is often considered in general workups for patients and their comorbidities though the 2020 European Society of Cardiology AF guidelines does recommend optimization of OSA for AF management.[8]

Mechanisms

OSA causes episodic hypoxia and increased intrathoracic pressures which via inflammation, oxidative stress and dysregulation of the autonomic nervous system, associate it with arrhythmia, hypertension, MACE, pulmonary hypertension and sudden cardiac death.[14,15] The cardiac autonomic nervous system is a key way the body maintains homeostasis of the heart and lungs. The sympathetic nervous system 'fight and flight' response and the parasympathetic

For peer review only

nervous system 'rest and digest' responses are antagonistic and imbalances or hyperactivity in both are linked to tachyarrhythmias, particularly AF and ventricular tachycardia (VT).[16] Abnormalities in breathing, especially apnoeic episodes, significantly elevate sympathetic activity and parasympathetic activity.[17] Pauses in breathing trigger parasympathetic activity via baroreceptors in the lung and cause paroxysmal bradycardias. This can reduce the atrial effective refractory period and promote increased activity in atrial myocytes in the pulmonary vein ostia, leading to AF. Studies in sleep apnoea patients show that their sympathetic activation is higher throughout the day and not just during sleep.[18] Increased sympathetic tone can also induce ectopy and increased firing in atrial tissue in the pulmonary veins, again, leading to AF. Parasympathetic and sympathetic activity can be measured using spectral analysis of heart rate variability (HRV) from 24-hour Holter recordings. Cycles of HRV are observed and grouped into ultra-low, very-low, low and high frequency bands. Loss of variability indicates higher risk of cardiovascular disease and arrhythmia. Analysis and comparison of HRV before CPAP initiation and a time after treatment will demonstrate any significant changes to cardiac autonomic control over this time. This can be correlated to arrhythmias identified on ILR. Heart rate turbulence (HRT) will also be derived from Holter data and analysed in the same way as HRV.

Mechanisms linking sleep apnoea and MACE include oxidative stress, endothelial dysfunction, mechanical stress and inflammation during hypoxic episodes in sleep apnoea.[19] The most researched markers associated with sleep apnoea were high sensitivity C-Reactive Protein (hs-CRP), tumour necrosis factor-alpha (TNF-α)[20] and interleukin-6 (IL-6)[21] identifying higher levels of inflammation in the body. A recent meta-analysis shows C-Reactive Protein (CRP), TNF-α and IL-6 reduce with CPAP treatment in patient with moderate to severe sleep apnoea[22]. However, all the studies involving TNF-α and IL-6 cited had low numbers of patients and further study with larger numbers is warranted. Other studies revealed matrix metalloproteinase-9 (MMP-9) is a useful marker in identifying hypertension in sleep apnoea patients. It is associated with oxidative stress and therefore cardiovascular damage and has been shown to be increased in

For peer review only

hypoxia.[23] Increased levels of MMP-9 have been correlated with increasing severity of sleep apnoea. High sensitivity Troponin-T (hsTnT) is heart specific enzyme which is released upon damage to the heart. N-terminal pro B-Type Natriuretic Peptide (NTproBNP) is a precursor to Brain Natriuretic Peptide (BNP) and is released from the walls of the heart in increased tension. Both are useful diagnostic and risk stratifying markers for ischaemia and heart failure respectively though there may be some overlap. HsTnT is also linked with increased incidence of AF. In a recent study Fibroblast Growth Factor 23 (FGF-23) and NTproBNP has been shown to correlate with the development of AF in the general population.[24] This marker may be an important link between sleep apnoea and AF. A better understanding of the arrhythmia profile, CPAP usage, autonomic dysfunction profile and biomarker profile may help identify what drives the cardiovascular mortality and morbidity in sleep apnoea patients and help target treatment.

Continuous Remote Monitoring and Other Monitoring

ILRs are small devices used to detect arrhythmias. Once implanted they continuously monitor the heart rhythm and record abnormalities for a period of 3 years or more. The data stored on the device is relayed via Bluetooth to a cellular-connected base-station or cellular phone which then sends the information to the database. Unlike Holter monitoring, they provide a complete extended temporal profile. CPAP usage is also recorded remotely allowing detailed sub-analysis of this cohort of patients to determine the effect of treatment. Additionally, measurement of cardiac autonomic function using Holter monitors at baseline and after 12 months of treatment gives another longitudinal view of the processes that could trigger arrhythmias in this group of patients. Identifying arrhythmia incidence in this group using an ILR device has only been performed in a small cohort of 20 patients[7] (specifically AF in this case) using the Medtronic Reveal XT Device. We plan on using the Medtronic Reveal LINQ which is smaller and less invasive and may improve participation and achieve greater recruitment. .This, alongside the biomarkers we are analysing at the same timepoints may elucidate the disparity between treated CPAP and continuing risk of heart disease and arrhythmia.

For peer review only

METHODS AND ANAYLYSIS

The Obstructive Sleep Apnoea and Cardiac Arrhythmia (OSCA) trial is a multi-hospital randomised nested cohort study. Participants are being recruited from sleep clinic after diagnosis of OSA and prior to CPAP initiation. All participants continue their usual treatment pathway for OSA alongside the trial protocol. A randomisation process occurs at consent, with a 1:1 split with one group receiving an ILR, 24-hour Holter, echocardiogram and blood tests, and the other receiving remote follow-up only; this follow-up is repeated in both groups at 12 months and 3 years remotely. The burden of arrhythmia in each group will be compared, with a focus on AF as defined by international guidelines.[8] Identification of arrhythmia and treatment is implemented by the principal investigators and guided by the Trial Steering Committee. The arrhythmia profile of those in the ILR group will be correlated to cardiac autonomic function parameters (both HRV and HRT), CPAP usage, echocardiogram findings and vascular biomarker profiles. MACE outcomes will be compared between the groups.

The OSCA trial started recruiting in October 2019 with an 18-month recruitment period and follow-up of 3 years. However, recruitment was halted very soon after due to the Covid-19 pandemic and has only recently restarted. We had recruited 31 participants (16 ILR, 15 no ILR) prior to the study being halted.

Objectives

The primary objectives are to characterize the arrhythmia incidence/burden in an OSA population using ILRs compared with standard care alone (the primary endpoint being clinically significant arrhythmias [of which we would treat]) and to assess cardiac autonomic function at baseline and following 12 months of CPAP therapy in the ILR group.

The secondary objectives are to: a) compare morbidity (with focus on MACE outcomes) of patients with OSA in both groups, b) explore frequency and onset of arrhythmias in the ILR-group vs no ILR-group with respect to CPAP usage profile, c) identify predictors for arrhythmia from all data gathered (including demographics, medical history and measured parameters as

For peer review only

above), d) characterize general cardiovascular and inflammatory biomarkers at initiation and during CPAP in the ILR group, e) explore OSA patients in terms of patient reported outcome measures in the ILR group.

Participants

The study population are those who have been diagnosed with moderate-severe OSA and are referred to start CPAP therapy. CPAP initiation clinics and trial procedures will be conducted at University Hospital Coventry & Warwickshire (UHCW), UK and Hospital of St. Cross, Rugby, UK.

Inclusion and Exclusion Criteria

Inclusions: Patients with moderate to severe OSA defined as an apnoea hypopnea index (AHI) of 15 or above, who require CPAP as standard care and are between the age of 18-75 years.

Exclusions: Patients who already have a diagnosis of AF/atrial flutter and/or ventricular tachycardia (VT), those with an ILR already in-situ or an established indication for an ILR and those with a palliative diagnosis i.e. life expectancy less than 3 years will be excluded from the trial. Patients who lack capacity cannot consent directly and are therefore excluded.

Participant pathway flow chart

Figure 1

Screening

Patients who have been newly diagnosed with OSA as an outpatient are identified from the sleep clinic and approached with an information leaflet. If interested, their suitability is assessed in person at clinic and they are enrolled into the study.

Eligibility and Baseline Assessment

Once initial consent is taken, baseline demographics, medical history and medication history is completed, and a 12-lead ECG is performed. Eligibility criteria are then checked and confirmed.

For peer review only

Randomization occurs at this point. If the participant is recruited into the ILR arm they have their ILR insertion, blood tests and questionnaires done. Those randomized to standard care alone will not need any further tests. Treatment pathways for OSA remain unchanged for both groups with their regular CPAP troubleshooting sessions continue (usually 1 month after initiation)

ILR-arm

The ILR insertion is performed by a trained practitioner in the left upper chest wall as per manufacturer guidelines, in this case REVEAL Linq (Medtronic, Minneapolis, USA). Observations and physical measurements are recorded and blood tests taken. Data from the ILR is uploaded on a 24-hour schedule so any clinically significant arrhythmias will be identified and treated promptly as per ESC guidelines. A 24 hour Holter monitor is applied between initial enrolment and ILR insertion. Data from the 24 hour Holter will then be analyzed for arrhythmia, HRV and HRT.

12-month follow-up

Participants in the ILR arm are contacted at 12 months with a 2 week window for repeat observations, physical measurements, blood tests and 24 hour Holter to be performed. Initial comparisons of 24hr Holter data, CPAP usage and arrhythmia detection by ILR can then be performed for each participant. Those in the standard care alone arm are contacted via telephone and a medical history is retaken to identify any incidence of arrhythmia. Their hospital record is then reviewed for any arrhythmia and CPAP usage since enrolment.

The Trial Steering Committee (TSC) will review any endpoints which are met and inform whether any interim analysis may be required.

3 year check and study end

For peer review only

At 3 years all participants are contacted via telephone to check their medical history. Any arrhythmias are documented. Those in the ILR arm are offered ILR removal. Participants are informed of the study end and any interim results will be offered to them. The planned data collection is summarised in Table 1.

Procedures	Consultant Sleep clinic/Sleep study	Baseline CPAP clinic	ECHO / / 24-hour tape$^{\beta}$	ILR if not done yet	12 months appointment$^{\beta}$	2 years	3 years
Screening	X						
Patient information sheet	X						
Eligibility assessment	X						
Informed consent		X					
Randomisation		X					
Medical history		X*			X$^{\beta}$	X	X
Clinical examination		X*					
Bedside observations		X*			X$^{\beta}$		
12-lead ECG†		X			X$^{\beta}$		
Health questionnaires (EQ5D) †		X			X$^{\beta}$		
Blood samples (Routine, NTpro-BNP, hsTnT, hsCRP IL-6, TNF-α, MMP-9, FGF-23)†		X			X$^{\beta}$		
Echocardiography - (LV/RV dimensions and functions and other ECHO parameters)‡			X$^{\beta}$		X$^{\beta}$		
24-hour Holter‡			X$^{\beta}$				
Standard therapy (lifestyle advice, referral to bariatric or weight loss services as usual)		X*					
Continuous Positive Airway Pressure check		X*					
Insertion of Injectable Loop Recorder (ILR) †		X		X			
ILR remote monitoring†		X					X
Adverse events (include SAEs - Hospital admissions)		X					X
Hospital readmissions and mortality		X					X
Arrhythmia detection and referral for treatment†		X					X
Removal of ILR							X

* Standard treatment pathway † For ILR group only ‡ For ILR group where available
β Research activities will be optional and conducted in line with trust COVID-19 safety policy subject to capacity and capability

Table .1 Schedule of Events

For peer review only

Patient and Public Involvement

Feedback and design input was sought from our local public and patient involvement groups on two occasions. The first was feedback on a preliminary patient information sheet (PIS) detailing the study. These perspectives changed the way the PIS was formatted and worded. It also streamlined the patient pathway to reduce the number of hospital visits.

The second session concluded with an agreement that a general patient would be happy to take part in the study as it would have additional monitoring benefits as well as screening them for other medical issues. They asked about how the research team would minimise extra patient travel and whether participants would receive any reimbursement for travel outside standard care. The Reveal-LINQ device safety was discussed, and the group felt that other than initial insertion it should not be an issue for participants.

A member of this group was asked to join the trial steering committee.

Power Calculation

The trial will recruit 200 participants based on AF incidence of 20%[7] using an ILR and 5% using traditional AF detection methods. This gives a 0.99 power with an alpha of 0.05 when comparing incidence of arrhythmia in the ILR arm compared with the standard care alone arm.

Statistical Analysis

For the primary objective of comparing arrhythmias detection rates between ILR group and standard care group, a chi-squared test or a Fisher's test will be used. For the co-primary objective of comparing mean HRT/HRV at baseline and 12 months in the ILR group, a paired t-test will be used. Mean difference and 95% confidence interval (CI) will also be reported.

For secondary objectives: a) compare MACE between both arms at follow up using a chi-squared test or a Fisher's exact test as appropriate. b) correlate arrhythmias detected in ILR at follow-up with effective CPAP usage using a chi-squared test or a Fisher's exact test as appropriate. c) for the ILR group, we will identify predictors for arrhythmia. To identify

For peer review only

predictors and with outcome being whether a patient has arrhythmia during the study (yes/no), we will fit simple and multiple logistic regression models. The explanatory variables in the logistic models will be the potential predictors for arrhythmia collected at baseline visit including age, gender, comorbidity, alcohol/smoking history, HRV and HRT parameters. d) compare vascular biomarkers before and after CPAP in the ILR arm using paired t-tests. e) perform longitudinal analysis of patient reported outcome measures. All tests will be performed at 5% significance level.

Conclusion

The OSCA trial will clarify the incidence of arrhythmias not readily identified using short-term cardiac monitoring. This in turn may help reduce the excess cardiovascular mortality that is known to exist in these patients despite CPAP therapy. Assessment of cardiac autonomic function may help identify mechanisms of this excess risk to help focus future studies.

ETHICS AND DISSEMINATION

Ethics

The Heath Research Authority and Solihull Research Ethics Committee (18//WM/0129) have approved the trial design and research protocol. All participants will have informed consent obtained in writing by an investigator. The main ethical considerations are to do with a the minimally invasive nature of ILR insertion which would be beyond standard care for OSA patients. This was discussed in detail at a patient and participant research advisory group meeting who felt it was appropriate for the type of research. It was noted that it was a short, minimally-invasive procedure and could potentially identify a heart rhythm problem early which would benefit the participant. Other considerations were additional blood tests and non-invasive monitoring which were deemed not to be a significant additional burden to participants due to only 2 additional visits beyond standard care.

Monitoring

For peer review only

The trial is being conducted in accordance with Good Clinical Practice, UK Law and the Declaration of Helsinki 2002. The TSC consists of sleep, respiratory and arrhythmia specialists, and a member from the Patients and Public Engagement committee. This group will meet annually to evaluate progress of the study in terms of recruitment, endpoints and adverse events. Publication of results will be overseen by the TSC. Protocol amendments will be submitted by the investigators with the sponsor to REC as required.

Dissemination and Data Statement

The full data set, study protocol and consent material will be anonymized and published in a suitable open data bank. We will aim to publish the primary objective of arrhythmia incidence as detected by ILR in a peer-reviewed journal with copies distributed to participants in the study and to the patient advisory group. Any findings relating to each individual will be shared with the relevant participant. We aim to publish details of arrhythmia burden related to cardiac autonomic function and CPAP usage in peer-reviewed journals.

For peer review only

AUTHOR CONTRIBUTIONS

Dr Hejie He – Primary author, Study Design

Dr Thomas Lachlan – Study design and reviewer

Dr Nakul Chandan – Study design and reviewer

Dr Ven Gee Lim – Study design and reviewer

Dr Peter Kimani – Statistical analysis

Professor Andre Ng- Study design and reviewer

Dr Asad Ali - Study design and reviewer, sleep lead/collaborator

Professor Harpal Randeva - Study design and reviewer, co-supervisor

Professor Faizel Osman – Primary supervisor Study Design and reviewer

Sponsor:

Research & Development, 4th Floor Rotunda
University Hospitals Coventry & Warwickshire NHS Trust
Clifford Bridge Road
Coventry
CV2 2DX
Email: ceri.jones@uhcw.nhs.uk

FUNDING STATEMENT

This work was supported by Medtronic grant number [ERP-2018-11257] and British Heart

Foundation grant number [PG/22/10931]

COMPETING INTERESTS STATEMENT

No author involved has any conflict of interest

For peer review only

REFERENCES

1. Davies, R. J. *et al.* The epidemiology sleep apnoea Introductory article. *Thorax* **51**, 65–70 (1996).

2. Punjabi, N. M. The epidemiology of adult obstructive sleep apnea. *Proc Am Thorac Soc* **5**, 136–43 (2008).

3. Benjafield, A. v. *et al.* Estimation of the global prevalence and burden of obstructive sleep apnoea: a literature-based analysis. *Lancet Respir Med* **7**, 687–698 (2019).

4. Selim, B. J. *et al.* The association between nocturnal cardiac arrhythmias and sleep-disordered breathing: The DREAM study. *Journal of Clinical Sleep Medicine* (2016) doi:10.5664/jcsm.5880.

5. Akar Bayram, N. *et al.* Prevalence of cardiac arrhythmia in obstructive sleep apnea syndrome. *Turk J Med Sci* **40**, 843–850 (2010).

6. Mehra, R. *et al.* Association of nocturnal arrhythmias with sleep-disordered breathing: The sleep heart health study. *Am J Respir Crit Care Med* **173**, 910–916 (2006).

7. Yeung, C. *et al.* Extended Cardiac Monitoring in Patients With Severe Sleep Apnea and No History of Atrial Fibrillation (The Reveal XT-SA Study). *American Journal of Cardiology* **122**, 1885–1889 (2018).

8. Hindricks, G. *et al. 2020 ESC Guidelines for the diagnosis and management of atrial fibrillation developed in collaboration with the European Association of Cardio-Thoracic Surgery (EACTS) The Task Force for the diagnosis and management of atrial fibrillation of the Europea*. (2020) doi:10.1093/eurheartj/ehaa612.

9. Marulanda-Londoño, E. & Chaturvedi, S. The Interplay between Obstructive Sleep Apnea and Atrial Fibrillation. *Front Neurol* **8**, 668 (2017).

For peer review only

10. Patil, S. P. *et al.* Treatment of adult obstructive sleep apnea with positive airway pressure: An American academy of sleep medicine systematic review, meta-analysis, and GRADE assessment. *Journal of Clinical Sleep Medicine* vol. 15 301–334 Preprint at https://doi.org/10.5664/jcsm.7638 (2019).

11. McEvoy, R. D. *et al.* CPAP for Prevention of Cardiovascular Events in Obstructive Sleep Apnea. *New England Journal of Medicine* **375**, 919–931 (2016).

12. Yu, J. *et al.* Association of Positive Airway Pressure With Cardiovascular Events and Death in Adults With Sleep Apnea. *JAMA* **318**, 156 (2017).

13. Drager, L. F., McEvoy, R. D., Barbe, F., Lorenzi-Filho, G. & Redline, S. Sleep apnea and cardiovascular disease: Lessons from recent trials and need for team science. *Circulation* **136**, 1840–1850 (2017).

14. Bradley, T. D. & Floras, J. S. Obstructive sleep apnoea and its cardiovascular consequences. *The Lancet* Preprint at https://doi.org/10.1016/S0140-6736(08)61622-0 (2009).

15. Somers, V. K. *et al.* Sleep Apnea and Cardiovascular Disease: An American Heart Association/American College of Cardiology Foundation Scientific Statement From the American Heart Association Council for High Blood Pressure Research Professional Education Committee, Council on . *Circulation* **118**, 1080–1111 (2008).

16. Shen, M. J. & Zipes, D. P. Role of the Autonomic Nervous System in Modulating Cardiac Arrhythmias This Review is in a thematic series on The Autonomic Nervous System and the Cardiovascular System, which includes the following articles: Role of the Autonomic Nervous System in Modula. *Circ Res* **114**, 1004–1021 (2014).

17. Khoo, M. C. K. & Blasi, A. Sleep-related Changes in Autonomic Control in Obstructive Sleep Apnea: A model-based perspective. *Respir Physiol Neurobiol* **188**, 267 (2013).

For peer review only

18. Somers, V. K., Dyken, M. E., Clary, M. P. & Abboud, F. M. Sympathetic neural mechanisms in obstructive sleep apnea. *J Clin Invest* **96**, 1897–904 (1995).

19. Javaheri, S. *et al.* Sleep Apnea: Types, Mechanisms, and Clinical Cardiovascular Consequences. *J Am Coll Cardiol* **69**, 841–858 (2017).

20. Wang, J. *et al.* Impact of Obstructive Sleep Apnea Syndrome on Endothelial Function, Arterial Stiffening, and Serum Inflammatory Markers: An Updated Meta-analysis and Metaregression of 18 Studies. *J Am Heart Assoc* **4**, (2015).

21. Imani, M. M. *et al.* Evaluation of Serum and Plasma Interleukin-6 Levels in Obstructive Sleep Apnea Syndrome: A Meta-Analysis and Meta-Regression. *Front Immunol* **11**, 1343 (2020).

22. Baessler, A. *et al.* Treatment for sleep apnea by continuous positive airway pressure improves levels of inflammatory markers - A meta-analysis. *Journal of Inflammation (United Kingdom)* **10**, 1–10 (2013).

23. Wang, S., Li, S., Wang, B., Liu, J. & Tang, Q. Matrix Metalloproteinase-9 Is a Predictive Factor for Systematic Hypertension and Heart Dysfunction in Patients with Obstructive Sleep Apnea Syndrome. *Biomed Res Int* **2018**, 1569701 (2018).

24. Navarro-García, J. A. *et al.* Fibroblast growth factor-23 promotes rhythm alterations and contractile dysfunction in adult ventricular cardiomyocytes. *Nephrology Dialysis Transplantation* (2019) doi:10.1093/ndt/gfy392.

FIGURE LEGEND

Figure 1. Patient Flow Diagram. *Holter monitor, Blood tests and Questionnaires

For peer review only

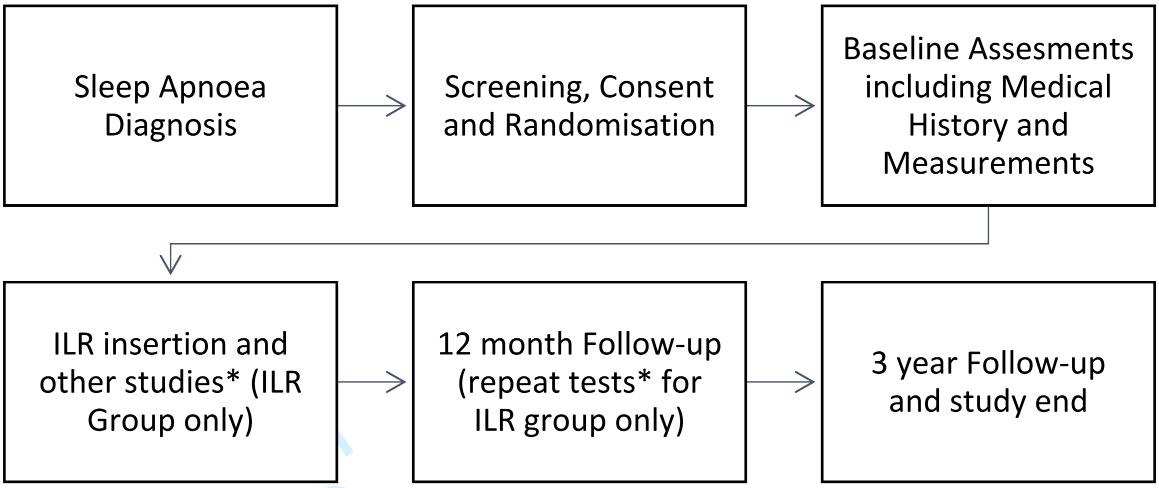

For peer review only

Reporting checklist for protocol of a clinical trial.

Based on the SPIRIT guidelines.

Instructions to authors

Complete this checklist by entering the page numbers from your manuscript where readers will find each of the items listed below.

Your article may not currently address all the items on the checklist. Please modify your text to include the missing information. If you are certain that an item does not apply, please write "n/a" and provide a short explanation.

Upload your completed checklist as an extra file when you submit to a journal.

In your methods section, say that you used the SPIRITreporting guidelines, and cite them as:

Chan A-W, Tetzlaff JM, Gøtzsche PC, Altman DG, Mann H, Berlin J, Dickersin K, Hróbjartsson A, Schulz KF, Parulekar WR, Krleža-Jerić K, Laupacis A, Moher D. SPIRIT 2013 Explanation and Elaboration: Guidance for protocols of clinical trials. BMJ. 2013;346:e7586

		Reporting Item	Page Number
**Administrative information**			
Title	#1	Descriptive title identifying the study design, population, interventions, and, if applicable, trial acronym	1
Trial registration	#2a	Trial identifier and registry name. If not yet registered,	3

For peer review only

		name of intended registry	
Trial registration: data set	#2b	All items from the World Health Organization Trial Registration Data Set	n/a
Protocol version	#3	Date and version identifier	2
Funding	#4	Sources and types of financial, material, and other support	2
Roles and responsibilities: contributorship	#5a	Names, affiliations, and roles of protocol contributors	17
Roles and responsibilities: sponsor contact information	#5b	Name and contact information for the trial sponsor	18
Roles and responsibilities: sponsor and funder	#5c	Role of study sponsor and funders, if any, in study design; collection, management, analysis, and interpretation of data; writing of the report; and the decision to submit the report for publication, including whether they will have ultimate authority over any of these activities	17-18
Roles and responsibilities: committees	#5d	Composition, roles, and responsibilities of the coordinating centre, steering committee, endpoint adjudication committee, data management team, and other individuals or groups overseeing the trial, if applicable (see Item 21a for data monitoring committee)	14

Introduction

For peer review only

Background and rationale	#6a	Description of research question and justification for undertaking the trial, including summary of relevant studies (published and unpublished) examining benefits and harms for each intervention	5
Background and rationale: choice of comparators	#6b	Explanation for choice of comparators	n/a
Objectives	#7	Specific objectives or hypotheses	9
Trial design	#8	Description of trial design including type of trial (eg, parallel group, crossover, factorial, single group), allocation ratio, and framework (eg, superiority, equivalence, non-inferiority, exploratory)	9
**Methods: Participants, interventions, and outcomes**			
Study setting	#9	Description of study settings (eg, community clinic, academic hospital) and list of countries where data will be collected. Reference to where list of study sites can be obtained	9
Eligibility criteria	#10	Inclusion and exclusion criteria for participants. If applicable, eligibility criteria for study centres and individuals who will perform the interventions (eg,	10

For peer review only

		surgeons, psychotherapists)	
Interventions: description	#11a	Interventions for each group with sufficient detail to allow replication, including how and when they will be administered	10
Interventions: modifications	#11b	Criteria for discontinuing or modifying allocated interventions for a given trial participant (eg, drug dose change in response to harms, participant request, or improving / worsening disease)	11
Interventions: adherance	#11c	Strategies to improve adherence to intervention protocols, and any procedures for monitoring adherence (eg, drug tablet return; laboratory tests)	n/a
Interventions: concomitant care	#11d	Relevant concomitant care and interventions that are permitted or prohibited during the trial	n/a
Outcomes	#12	Primary, secondary, and other outcomes, including the specific measurement variable (eg, systolic blood pressure), analysis metric (eg, change from baseline, final value, time to event), method of aggregation (eg, median, proportion), and time point for each outcome. Explanation of the clinical relevance of chosen efficacy and harm outcomes is strongly recommended	9
Participant timeline	#13	Time schedule of enrolment, interventions (including any run-ins and washouts), assessments, and visits for participants. A schematic diagram is highly recommended (see Figure)	12

For peer review only

Sample size	#14	Estimated number of participants needed to achieve study objectives and how it was determined, including clinical and statistical assumptions supporting any sample size calculations	13
Recruitment	#15	Strategies for achieving adequate participant enrolment to reach target sample size	10
**Methods: Assignment of interventions (for controlled trials)**			
Allocation: sequence generation	#16a	Method of generating the allocation sequence (eg, computer-generated random numbers), and list of any factors for stratification. To reduce predictability of a random sequence, details of any planned restriction (eg, blocking) should be provided in a separate document that is unavailable to those who enrol participants or assign interventions	11
Allocation concealment mechanism	#16b	Mechanism of implementing the allocation sequence (eg, central telephone; sequentially numbered, opaque, sealed envelopes), describing any steps to conceal the sequence until interventions are assigned	11
Allocation: implementation	#16c	Who will generate the allocation sequence, who will enrol participants, and who will assign participants to interventions	11

For peer review only

Blinding (masking)	#17a	Who will be blinded after assignment to interventions (eg, trial participants, care providers, outcome assessors, data analysts), and how	n/a
Blinding (masking): emergency unblinding	#17b	If blinded, circumstances under which unblinding is permissible, and procedure for revealing a participant's allocated intervention during the trial	n/a
**Methods: Data collection, management, and analysis**			
Data collection plan	#18a	Plans for assessment and collection of outcome, baseline, and other trial data, including any related processes to promote data quality (eg, duplicate measurements, training of assessors) and a description of study instruments (eg, questionnaires, laboratory tests) along with their reliability and validity, if known. Reference to where data collection forms can be found, if not in the protocol	14
Data collection plan: retention	#18b	Plans to promote participant retention and complete follow-up, including list of any outcome data to be collected for participants who discontinue or deviate from intervention protocols	14
Data management	#19	Plans for data entry, coding, security, and storage, including any related processes to promote data quality (eg, double data entry; range checks for data values).	14

For peer review only

		Reference to where details of data management procedures can be found, if not in the protocol	
Statistics: outcomes	#20a	Statistical methods for analysing primary and secondary outcomes. Reference to where other details of the statistical analysis plan can be found, if not in the protocol	13
Statistics: additional analyses	#20b	Methods for any additional analyses (eg, subgroup and adjusted analyses)	13
Statistics: analysis population and missing data	#20c	Definition of analysis population relating to protocol non-adherence (eg, as randomised analysis), and any statistical methods to handle missing data (eg, multiple imputation)	13
**Methods: Monitoring**			
Data monitoring: formal committee	#21a	Composition of data monitoring committee (DMC); summary of its role and reporting structure; statement of whether it is independent from the sponsor and competing interests; and reference to where further details about its charter can be found, if not in the protocol. Alternatively, an explanation of why a DMC is not needed	n/a
Data monitoring: interim analysis	#21b	Description of any interim analyses and stopping guidelines, including who will have access to these interim results and make the final decision to terminate the trial	n/a
Harms	#22	Plans for collecting, assessing, reporting, and managing solicited and spontaneously reported adverse events and other unintended effects of trial interventions or trial	14

For peer review only

		conduct	
Auditing	#23	Frequency and procedures for auditing trial conduct, if any, and whether the process will be independent from investigators and the sponsor	14
**Ethics and dissemination**			
Research ethics approval	#24	Plans for seeking research ethics committee / institutional review board (REC / IRB) approval	14
Protocol amendments	#25	Plans for communicating important protocol modifications (eg, changes to eligibility criteria, outcomes, analyses) to relevant parties (eg, investigators, REC / IRBs, trial participants, trial registries, journals, regulators)	14
Consent or assent	#26a	Who will obtain informed consent or assent from potential trial participants or authorised surrogates, and how (see Item 32)	14
Consent or assent: ancillary studies	#26b	Additional consent provisions for collection and use of participant data and biological specimens in ancillary studies, if applicable	n/a
Confidentiality	#27	How personal information about potential and enrolled participants will be collected, shared, and maintained in order to protect confidentiality before, during, and after the trial	14
Declaration of	#28	Financial and other competing interests for principal	18

For peer review only

interests		investigators for the overall trial and each study site	
Data access	#29	Statement of who will have access to the final trial dataset, and disclosure of contractual agreements that limit such access for investigators	14
Ancillary and post trial care	#30	Provisions, if any, for ancillary and post-trial care, and for compensation to those who suffer harm from trial participation	11
Dissemination policy: trial results	#31a	Plans for investigators and sponsor to communicate trial results to participants, healthcare professionals, the public, and other relevant groups (eg, via publication, reporting in results databases, or other data sharing arrangements), including any publication restrictions	14
Dissemination policy: authorship	#31b	Authorship eligibility guidelines and any intended use of professional writers	n/a
Dissemination policy: reproducible research	#31c	Plans, if any, for granting public access to the full protocol, participant-level dataset, and statistical code	14
**Appendices**			
Informed consent materials	#32	Model consent form and other related documentation given to participants and authorised surrogates	14
Biological specimens	#33	Plans for collection, laboratory evaluation, and storage of biological specimens for genetic or molecular analysis in the current trial and for future use in ancillary studies, if applicable	n/a

For peer review only

The SPIRIT Explanation and Elaboration paper is distributed under the terms of the Creative Commons Attribution License CC-BY-NC. This checklist was completed on 06. December 2022 using https://www.goodreports.org/, a tool made by the EQUATOR Network in collaboration with Penelope.ai

For peer review only

BMJ Open

PROTOCOL: Obstructive Sleep Apnoea and Cardiac Arrhythmias (OSCA) Trial – A nested cohort study using Injectable Loop Recorders and Holter monitoring in Obstructive Sleep Apnoea Patients

Journal:	*BMJ Open*
Manuscript ID	bmjopen-2022-070884.R1
Article Type:	Protocol
Date Submitted by the Author:	13-Jan-2023
Complete List of Authors:	He, Hejie; University Hospitals Coventry and Warwickshire NHS Trust, Cardiology; University of Warwick Warwick Medical School, Warwick Medical School<br>Lachlan, Thomas; University Hospitals Coventry and Warwickshire NHS Trust, Cardiology; University of Warwick Warwick Medical School,<br>Chandan, Nakul; University Hospitals Coventry and Warwickshire NHS Trust, Cardiology<br>Lim, Ven; University Hospitals Coventry and Warwickshire NHS Trust, Cardiology; University of Warwick Warwick Medical School, Warwick Medical School<br>Kimani, Peter; University of Warwick Faculty of Medicine, Warwick Medical School<br>Ng, G Andre; University of Leicester, Cardiovascular Sciences<br>Ali, Asad; University Hospitals Coventry and Warwickshire NHS Trust, Cardiology<br>Randeva, Harpal; University Hospitals Coventry and Warwickshire NHS Trust, Cardiology; University of Warwick Warwick Medical School, Warwick Medical School<br>Osman, Faizel; University Hospitals Coventry and Warwickshire NHS Trust, Cardiology; University of Warwick Warwick Medical School,
**Primary Subject Heading**:	Cardiovascular medicine
Secondary Subject Heading:	Respiratory medicine
Keywords:	Pacing & electrophysiology < CARDIOLOGY, Adult cardiology < CARDIOLOGY, Adult thoracic medicine < THORACIC MEDICINE, SLEEP MEDICINE

SCHOLARONE™
Manuscripts

PROTOCOL: Obstructive Sleep Apnoea and Cardiac Arrhythmias (OSCA) Trial – A nested cohort study using Injectable Loop Recorders and Holter monitoring in Obstructive Sleep Apnoea Patients

Authors:

Hejie He MRCP[1]
Cardiology Research Fellow

Thomas Lachlan MRCP[1]
Cardiology Research Fellow

Nakul Chandan MRCP[1]
Cardiology Research Fellow

Ven Lim MRCP PhD[1]
Cardiology Clinical lecturer

Peter K Kimani BSc MSc PhD[2]
Associate Professor in Medical Statistics

G Andre Ng PhD, FRCP, FEHRA[3]
Professor of Cardiac Electrophysiology
Consultant Cardiologist / Electrophysiologist

Asad Ali MD FRCP[1]
Consultant Sleep and Respiratory Physician
Lead for Sleep Medicine

Harpal S Randeva FRCP, FAcadTM, PhD[4,5]
Director of R&D, Professor of Medicine
Head & Clinical Lead for Diabetes, Endocrinology & Metabolism

Faizel Osman MD FRCP FESC[1,2]
Consultant Cardiologist /Electrophysiologist
Honorary Professor of Cardiology
Director of UHCW R&D Cardio-Metabolic Institute

Address:

[1]Department of Cardiology
University Hospital Coventry
Clifford Bridge Road
Coventry
CV2 2DX
United Kingdom

[2]Warwick Medical School
University of Warwick
Gibbet Hill Road
Coventry CV4 7HL

For peer review only

United Kingdom

[3]Department of Cardiovascular Sciences
University of Leicester
NIHR Leicester Biomedical Research Centre
Glenfield Hospital Leicester
LE3 9QP

[4]Warwickshire Institute for the Study of Diabetes, Endocrinology and Metabolism (WISDEM), University Hospitals Coventry and Warwickshire NHS Trust, Coventry,
CV2 2DX
United Kingdom

[5]Aston Medical School, College of Health and Life Sciences, Aston University, Birmingham
B4 7ET
United Kingdom

Corresponding author: Professor Faizel Osman
Tel: +44 (0) 2476 965668
Email: faizel.osman@uhcw.nhs.uk
Abstract Word count: 299
Text Word Count: 2887
Conflicts of interest: none

Keywords: CPAP, Obstructive Sleep Apnoea, Arrhythmia, Heart Rate Variability, Atrial Fibrillation, Cardiovascular Biomarkers

Protocol version: 2.1
Protocol Date: 27/01/2021

Funding: British Heart Foundation PG/22/10931
Medtronic ERP-2018-11257

For peer review only

ABSTRACT

Introduction: Obstructive sleep apnoea (OSA) is associated with increased cardiovascular morality despite continuous airways pressure (CPAP) therapy. This excess risk may be related to increased arrhythmia risk, especially atrial fibrillation (AF). The true incidence of arrhythmia in OSA patients is unknown. Implantable loop recorders (ILR) are powerful tools for detecting arrhythmias long-term. Cardiac autonomic function may be important in arrhythmogenesis in these patients but needs further study. We aim to identify the true incidence of arrhythmias (especially AF) using ILRs, assess cardiac autonomic function using Holter monitors in OSA patients and explore cardiovascular outcomes.

Methods and Analysis: A two-centre (University Hospital Coventry and St. Cross Hospital, Rugby) randomised controlled trial using Reveal LINQ (Medtronic, UK) ILR to identify precise arrhythmia (atrial/ventricular) incidence in moderate-severe OSA patients. 200 patients will be randomised 1:1 to standard care alone or standard care + ILR (+Holter monitor at baseline and 12months). The primary objective is to compare arrhythmia detection over 3years between the two groups. Cardiac autonomic function will be assessed in the ILR-arm at baseline and 12-months post CPAP. Secondary objectives will explore the mechanisms linking OSA and arrhythmia using cardiac autonomic function parameters based on Holter recordings and circulating biomarkers (hs-TnT, NT-proBNP, MMP-9, FGF-23, hs-CRP IL-6, TNF-α) before and after CPAP initiation in the ILR-arm.

Ethics and Dissemination: This study has been approved by the Health Research Authority after examination by the Solihull Research and Ethics Committee. The main ethical considerations was the minimally invasive nature of ILR insertion outside of usual care. Patient advisory groups were consulted with a positive outcome for this type of research. We plan on publishing papers in peer-reviewed journals based on the primary objective and any interesting findings from secondary objectives. We will endeavour to publish all relevant data.

Registration: ClinicalTrials.gov (NCT03866148) and EudraCT (2019-002995-15).

For peer review only

Strengths and limitations of this study

- OSCA uses implantable loop recorders which are the gold standard in arrhythmia detection thereby determining the incidence of arrhythmias over 3 years in this obstructive sleep apnoea population.
- The combination of cardiac autonomic function surrogates in heart rate variability, heart rate turbulence with biomarkers and arrhythmia burden using the ILR is novel and likely to be hypothesis generating.
- As there are few exclusion criteria, the external validity of this study is likely to be high.
- Due to the nature of nested cohort studies, the causation of arrhythmias cannot be determined.

For peer review only

INTRODUCTION

Obstructive Sleep Apnoea and Arrhythmias

Obstructive sleep apnoea (OSA) is characterised by abnormal or cessation of breathing during sleep due to narrowing or closure of the upper airways. The estimated prevalence of moderate or severe disease ranges from 5 to 50% with the highest proportion seemingly in overweight middle-aged men.[1–3] These patients suffer from increased snoring, excessive daytime sleepiness and a higher incidence of cardiovascular disease and arrhythmia, even after controlling for weight and comorbidities (such as diabetes and hypertension) which are prevalent in both OSA and cardiovascular disease. Arrhythmias can compound symptoms of shortness of breath and fatigue in OSA patients and can lead to stroke, sudden cardiac death (SCD) and heart failure. Increased severity of OSA increases risk of arrhythmia, the most common being atrial fibrillation (AF) with up to 60% of patients with sleep apnoea having some type of arrhythmia.[4,5] Some studies of OSA patients suggest AF in 4.8%[6] yet one study using an implantable loop recorder (ILR) demonstrated AF in 20% (up to 5 times higher than the general population).[7] Other arrhythmias including heart block, bradycardias and ventricular tachycardia also appear to be more prevalent. Despite this, European guidelines do not specifically encourage screening for sleep apnoea in AF.[8] Similarly, AF and other arrhythmias are not routinely screened for in OSA patients.[9]

Continuous positive airways pressure (CPAP) is the mainstay of hospital prescribed treatment. This gives airways support to a patient overnight via a mask or nasal prongs and is proven to improve patient symptoms and the number of hypopnoea and apnoeic episodes.[10] Its effect on associated morbidity and mortality suggest it reduces episodes of arrhythmia and major cardiovascular events (MACE [defined as death, myocardial infarction, coronary revascularisation, stroke and heart failure]). However, a recent meta-analysis and the SAVE trial involving 2,687 patients in the primary analysis demonstrated no significant reduction in

For peer review only

cardiovascular outcomes or reduction in atrial fibrillation.[11,12] Further study is needed to determine what factors prevent CPAP from reducing this morbidity and mortality.[13]

Arrhythmias, most commonly AF, are detected by ECGs or ambulatory ECG monitoring (e.g. Holter monitoring). However, these diagnostic tools can often miss significant paroxysmal arrhythmias. Short paroxysms of arrhythmia not detected by standard screening could account for a proportion of the increased morbidity and mortality despite CPAP therapy seen in previous studies. Whilst shown to be effective at near eliminating apnoeas and hypopnoeas, CPAP therapy can be heterogenous in its usage with mean duration of treatment and mask types varying between studies. Underusage of CPAP or differences in therapy types (e.g. nasal CPAP vs full-mask CPAP) and therefore incomplete treatment may also contribute. Other less studied mechanisms that link OSA with MACE and arrhythmia could also explain this disparity but need further study.

Current Guidelines

OSA is an independent risk factor for arrhythmia though there is no clear consensus on the overall incidence and mechanisms for this. Current guidelines do not encourage screening for arrhythmia in OSA and vice versa though it is often considered in general workups for patients and their comorbidities though the 2020 European Society of Cardiology AF guidelines does recommend optimization of OSA for AF management.[8]

Mechanisms

OSA causes episodic hypoxia and increased intrathoracic pressures which via inflammation, oxidative stress and dysregulation of the autonomic nervous system, associate it with arrhythmia, hypertension, MACE, pulmonary hypertension and sudden cardiac death.[14,15] The cardiac autonomic nervous system is a key way the body maintains homeostasis of the heart and lungs. The sympathetic nervous system 'fight and flight' response and the parasympathetic

For peer review only

nervous system 'rest and digest' responses are antagonistic and imbalances or hyperactivity in both are linked to tachyarrhythmias, particularly AF and ventricular tachycardia (VT).[16] Abnormalities in breathing, especially apnoeic episodes, significantly elevate sympathetic activity and parasympathetic activity.[17] Pauses in breathing trigger parasympathetic activity via baroreceptors in the lung and cause paroxysmal bradycardias. This can reduce the atrial effective refractory period and promote increased activity in atrial myocytes in the pulmonary vein ostia, leading to AF. Studies in sleep apnoea patients show that their sympathetic activation is higher throughout the day and not just during sleep.[18] Increased sympathetic tone can also induce ectopy and increased firing in atrial tissue in the pulmonary veins, again, leading to AF. Parasympathetic and sympathetic activity can be measured using spectral analysis of heart rate variability (HRV) from 24-hour Holter recordings. Cycles of HRV are observed and grouped into ultra-low, very-low, low and high frequency bands. Loss of variability indicates higher risk of cardiovascular disease and arrhythmia. Analysis and comparison of HRV before CPAP initiation and a time after treatment will demonstrate any significant changes to cardiac autonomic control over this time. This can be correlated to arrhythmias identified on ILR. Heart rate turbulence (HRT) will also be derived from Holter data and analysed in the same way as HRV.

Mechanisms linking sleep apnoea and MACE include oxidative stress, endothelial dysfunction, mechanical stress and inflammation during hypoxic episodes in sleep apnoea.[19] The most researched markers associated with sleep apnoea were high sensitivity C-Reactive Protein (hs-CRP), tumour necrosis factor-alpha (TNF-α)[20] and interleukin-6 (IL-6)[21] identifying higher levels of inflammation in the body. A recent meta-analysis shows C-Reactive Protein (CRP), TNF-α and IL-6 reduce with CPAP treatment in patient with moderate to severe sleep apnoea[22]. However, all the studies involving TNF-α and IL-6 cited had low numbers of patients and further study with larger numbers is warranted. Other studies revealed matrix metalloproteinase-9 (MMP-9) is a useful marker in identifying hypertension in sleep apnoea patients. It is associated with oxidative stress and therefore cardiovascular damage and has been shown to be increased in

For peer review only

hypoxia.[23] Increased levels of MMP-9 have been correlated with increasing severity of sleep apnoea. High sensitivity Troponin-T (hsTnT) is heart specific enzyme which is released upon damage to the heart. N-terminal pro B-Type Natriuretic Peptide (NTproBNP) is a precursor to Brain Natriuretic Peptide (BNP) and is released from the walls of the heart in increased tension. Both are useful diagnostic and risk stratifying markers for ischaemia and heart failure respectively though there may be some overlap. HsTnT is also linked with increased incidence of AF. In a recent study Fibroblast Growth Factor 23 (FGF-23) and NTproBNP has been shown to correlate with the development of AF in the general population.[24] This marker may be an important link between sleep apnoea and AF. A better understanding of the arrhythmia profile, CPAP usage, autonomic dysfunction profile and biomarker profile may help identify what drives the cardiovascular mortality and morbidity in sleep apnoea patients and help target treatment.

Continuous Remote Monitoring and Other Monitoring

ILRs are small devices used to detect arrhythmias. Once implanted they continuously monitor the heart rhythm and record abnormalities for a period of 3 years or more. The data stored on the device is relayed via Bluetooth to a cellular-connected base-station or cellular phone which then sends the information to the database. Unlike Holter monitoring, they provide a complete extended temporal profile. CPAP usage is also recorded remotely allowing detailed sub-analysis of this cohort of patients to determine the effect of treatment. Additionally, measurement of cardiac autonomic function using Holter monitors at baseline and after 12 months of treatment gives another longitudinal view of the processes that could trigger arrhythmias in this group of patients. Identifying arrhythmia incidence in this group using an ILR device has only been performed in a small cohort of 20 patients[7] (specifically AF in this case) using the Medtronic Reveal XT Device. We plan on using the Medtronic Reveal LINQ which is smaller and less invasive and may improve participation and achieve greater recruitment. .This, alongside the biomarkers we are analysing at the same timepoints may elucidate the disparity between treated CPAP and continuing risk of heart disease and arrhythmia.

For peer review only

METHODS AND ANAYLYSIS

The Obstructive Sleep Apnoea and Cardiac Arrhythmia (OSCA) trial is a multi-hospital randomised nested cohort study. Participants are being recruited from sleep clinic after diagnosis of OSA and prior to CPAP initiation. All participants continue their usual treatment pathway for OSA alongside the trial protocol. A randomisation process occurs at consent, with a 1:1 split with one group receiving an ILR, 24-hour Holter, echocardiogram and blood tests, and the other receiving remote follow-up only; this follow-up is repeated in both groups at 12 months and 3 years remotely. The burden of arrhythmia in each group will be compared, with a focus on AF as defined by international guidelines.[8] Identification of arrhythmia and treatment is implemented by the principal investigators and guided by the Trial Steering Committee. The arrhythmia profile of those in the ILR group will be correlated to cardiac autonomic function parameters (both HRV and HRT), CPAP usage, echocardiogram findings and vascular biomarker profiles. MACE outcomes will be compared between the groups.

The OSCA trial started recruiting in October 2019 with an 18-month recruitment period and follow-up of 3 years. However, recruitment was halted very soon after due to the Covid-19 pandemic and has only recently restarted. We had recruited 31 participants (16 ILR, 15 no ILR) prior to the study being halted.

Objectives

The primary objectives are to characterize the arrhythmia incidence/burden in an OSA population using ILRs compared with standard care alone (the primary endpoint being clinically significant arrhythmias [of which we would treat]) and to assess cardiac autonomic function at baseline and following 12 months of CPAP therapy in the ILR group.

The secondary objectives are to: a) compare morbidity (with focus on MACE outcomes) of patients with OSA in both groups, b) explore frequency and onset of arrhythmias in the ILR-group vs no ILR-group with respect to CPAP usage profile, c) identify predictors for arrhythmia from all data gathered (including demographics, medical history and measured parameters as

For peer review only

above), d) characterize general cardiovascular and inflammatory biomarkers at initiation and during CPAP in the ILR group, e) explore OSA patients in terms of patient reported outcome measures in the ILR group.

Participants

The study population are those who have been diagnosed with moderate-severe OSA and are referred to start CPAP therapy. CPAP initiation clinics and trial procedures will be conducted at University Hospital Coventry & Warwickshire (UHCW), UK and Hospital of St. Cross, Rugby, UK.

Inclusion and Exclusion Criteria

Inclusions: Patients with moderate to severe OSA defined as an apnoea hypopnea index (AHI) of 15 or above, who require CPAP as standard care and are between the age of 18-75 years.

Exclusions: Patients who already have a diagnosis of AF/atrial flutter and/or ventricular tachycardia (VT), those with an ILR already in-situ or an established indication for an ILR and those with a palliative diagnosis i.e. life expectancy less than 3 years will be excluded from the trial. Patients who lack capacity cannot consent directly and are therefore excluded.

Participant pathway flow chart

Figure 1

Screening

Patients who have been newly diagnosed with OSA as an outpatient are identified from the sleep clinic and approached with an information leaflet. If interested, their suitability is assessed in person at clinic and they are enrolled into the study.

Eligibility and Baseline Assessment

Once initial consent is taken, baseline demographics, medical history and medication history is completed, and a 12-lead ECG is performed. Eligibility criteria are then checked and confirmed.

For peer review only

Randomization occurs at this point. If the participant is recruited into the ILR arm they have their ILR insertion, blood tests and questionnaires done. Those randomized to standard care alone will not need any further tests. Treatment pathways for OSA remain unchanged for both groups with their regular CPAP troubleshooting sessions continue (usually 1 month after initiation)

ILR-arm

The ILR insertion is performed by a trained practitioner in the left upper chest wall as per manufacturer guidelines, in this case REVEAL Linq (Medtronic, Minneapolis, USA). Observations and physical measurements are recorded and blood tests taken. Data from the ILR is uploaded on a 24-hour schedule so any clinically significant arrhythmias will be identified and treated promptly as per ESC guidelines. A 24 hour Holter monitor is applied between initial enrolment and ILR insertion. Data from the 24 hour Holter will then be analyzed for arrhythmia, HRV and HRT.

12-month follow-up

Participants in the ILR arm are contacted at 12 months with a 2 week window for repeat observations, physical measurements, blood tests and 24 hour Holter to be performed. Initial comparisons of 24hr Holter data, CPAP usage and arrhythmia detection by ILR can then be performed for each participant. Those in the standard care alone arm are contacted via telephone and a medical history is retaken to identify any incidence of arrhythmia. Their hospital record is then reviewed for any arrhythmia and CPAP usage since enrolment.

The Trial Steering Committee (TSC) will review any endpoints which are met and inform whether any interim analysis may be required.

3 year check and study end

For peer review only

At 3 years all participants are contacted via telephone to check their medical history. Any arrhythmias are documented. Those in the ILR arm are offered ILR removal. Participants are informed of the study end and any interim results will be offered to them. The planned data collection is summarised in Table 1.

Procedures	Consultant Sleep clinic/Sleep study	Baseline CPAP clinic	ECHO / / 24-hour tape$^{\beta}$	ILR if not done yet	12 months appointment$^{\beta}$	2 years	3 years
Screening	X						
Patient information sheet	X						
Eligibility assessment	X						
Informed consent		X					
Randomisation		X					
Medical history		X*			X^{β}	X	X
Clinical examination		X*					
Bedside observations		X*			X^{β}		
12-lead ECG†		X			X^{β}		
Health questionnaires (EQ5D) †		X			X^{β}		
Blood samples (Routine, NTpro-BNP, hsTnT, hsCRP IL-6, TNF-α, MMP-9, FGF-23)†		X			X^{β}		
Echocardiography - (LV/RV dimensions and functions and other ECHO parameters)‡			X^{β}		X^{β}		
24-hour Holter‡			X^{β}				
Standard therapy (lifestyle advice, referral to bariatric or weight loss services as usual)		X*					
Continuous Positive Airway Pressure check		X*					
Insertion of Injectable Loop Recorder (ILR) †		X		X			
ILR remote monitoring†		X					X
Adverse events (include SAEs - Hospital admissions)		X					X
Hospital readmissions and mortality		X					X
Arrhythmia detection and referral for treatment†		X					X
Removal of ILR							X

* Standard treatment pathway † For ILR group only ‡ For ILR group where available
β Research activities will be optional and conducted in line with trust COVID-19 safety policy subject to capacity and capability

Table .1 Schedule of Events

For peer review only

Patient and Public Involvement

Feedback and design input was sought from our local public and patient involvement groups on two occasions. The first was feedback on a preliminary patient information sheet (PIS) detailing the study. These perspectives changed the way the PIS was formatted and worded. It also streamlined the patient pathway to reduce the number of hospital visits.

The second session concluded with an agreement that a general patient would be happy to take part in the study as it would have additional monitoring benefits as well as screening them for other medical issues. They asked about how the research team would minimise extra patient travel and whether participants would receive any reimbursement for travel outside standard care. The Reveal-LINQ device safety was discussed, and the group felt that other than initial insertion it should not be an issue for participants.

A member of this group was asked to join the trial steering committee.

Power Calculation

The trial will recruit 200 participants based on AF incidence of 20%[7] using an ILR and 5% using traditional AF detection methods. This gives a 0.99 power with an alpha of 0.05 when comparing incidence of arrhythmia in the ILR arm compared with the standard care alone arm.

Statistical Analysis

For the primary objective of comparing arrhythmias detection rates between ILR group and standard care group, a chi-squared test or a Fisher's test will be used. For the co-primary objective of comparing mean HRT/HRV at baseline and 12 months in the ILR group, a paired t-test will be used. Mean difference and 95% confidence interval (CI) will also be reported.

For secondary objectives: a) compare MACE between both arms at follow up using a chi-squared test or a Fisher's exact test as appropriate. b) correlate arrhythmias detected in ILR at follow-up with effective CPAP usage using a chi-squared test or a Fisher's exact test as appropriate. c) for the ILR group, we will identify predictors for arrhythmia. To identify

For peer review only

predictors and with outcome being whether a patient has arrhythmia during the study (yes/no), we will fit simple and multiple logistic regression models. The explanatory variables in the logistic models will be the potential predictors for arrhythmia collected at baseline visit including age, gender, comorbidity, alcohol/smoking history, HRV and HRT parameters. d) compare vascular biomarkers before and after CPAP in the ILR arm using paired t-tests. e) perform longitudinal analysis of patient reported outcome measures. All tests will be performed at 5% significance level.

ETHICS AND DISSEMINATION

Ethics

The Heath Research Authority and Solihull Research Ethics Committee (18//WM/0129) have approved the trial design and research protocol. All participants will have informed consent obtained in writing by an investigator. The main ethical considerations are to do with a the minimally invasive nature of ILR insertion which would be beyond standard care for OSA patients. This was discussed in detail at a patient and participant research advisory group meeting who felt it was appropriate for the type of research. It was noted that it was a short, minimally-invasive procedure and could potentially identify a heart rhythm problem early which would benefit the participant. Other considerations were additional blood tests and non-invasive monitoring which were deemed not to be a significant additional burden to participants due to only 2 additional visits beyond standard care.

Monitoring

The trial is being conducted in accordance with Good Clinical Practice, UK Law and the Declaration of Helsinki 2002. The TSC consists of sleep, respiratory and arrhythmia specialists, and a member from the Patients and Public Engagement committee. This group will meet annually to evaluate progress of the study in terms of recruitment, endpoints and adverse events. Publication of results will be overseen by the TSC. Protocol amendments will be submitted by the investigators with the sponsor to REC as required.

For peer review only

Dissemination and Data Statement

The full data set, study protocol and consent material will be anonymized and published in a suitable open data bank. We will aim to publish the primary objective of arrhythmia incidence as detected by ILR in a peer-reviewed journal with copies distributed to participants in the study and to the patient advisory group. Any findings relating to each individual will be shared with the relevant participant. We aim to publish details of arrhythmia burden related to cardiac autonomic function and CPAP usage in peer-reviewed journals.

For peer review only

AUTHOR CONTRIBUTIONS

The OSCA research study was conceived by Faizel Osman, Thomas Lachlan and Hejie He. There were significant contributions in the design of the study by Asad Ali, Andre Ng and Harpal Randeva, particularly with the recruitment method, access to the necessary resources and outcome measures after conception. Peter Kimani reviewed the study design and performed power calculations for the primary outcomes as well as developing the statistical analysis methodology with Hejie He, Thomas Lachlan, Ven Gee Lim and Faizel Osman. Nakul Chandan, Thomas Lachlan and Hejie He have been the primary contributors to the day to day running of the trial and data acquisition so far whilst Faizel Osman and Harpal Randeva have been supervising. All authors have reviewed this manuscript and suggested changes as well reviewing the protocol and have approved this for submission. All authors have agreed their accountability for this manuscript.

Sponsor:

Research & Development, 4th Floor Rotunda
University Hospitals Coventry & Warwickshire NHS Trust
Clifford Bridge Road
Coventry
CV2 2DX
Email: ceri.jones@uhcw.nhs.uk

FUNDING STATEMENT

This work was supported by Medtronic grant number [ERP-2018-11257] and British Heart Foundation grant number [PG/22/10931]

COMPETING INTERESTS STATEMENT

Whilst Medtronic have provided some funding for staff costs and ILR devices, this has been directed through UHCW NHS Trust as the sponsor of the trial. Medtronic have not had any direct involvement in the design of the study or it’s conduct. There are no competing interests to state.

For peer review only

REFERENCES

1. Davies, R. J. *et al.* The epidemiology sleep apnoea Introductory article. *Thorax* **51**, 65–70 (1996).

2. Punjabi, N. M. The epidemiology of adult obstructive sleep apnea. *Proc Am Thorac Soc* **5**, 136–43 (2008).

3. Benjafield, A. v. *et al.* Estimation of the global prevalence and burden of obstructive sleep apnoea: a literature-based analysis. *Lancet Respir Med* **7**, 687–698 (2019).

4. Selim, B. J. *et al.* The association between nocturnal cardiac arrhythmias and sleep-disordered breathing: The DREAM study. *Journal of Clinical Sleep Medicine* (2016) doi:10.5664/jcsm.5880.

5. Akar Bayram, N. *et al.* Prevalence of cardiac arrhythmia in obstructive sleep apnea syndrome. *Turk J Med Sci* **40**, 843–850 (2010).

6. Mehra, R. *et al.* Association of nocturnal arrhythmias with sleep-disordered breathing: The sleep heart health study. *Am J Respir Crit Care Med* **173**, 910–916 (2006).

7. Yeung, C. *et al.* Extended Cardiac Monitoring in Patients With Severe Sleep Apnea and No History of Atrial Fibrillation (The Reveal XT-SA Study). *American Journal of Cardiology* **122**, 1885–1889 (2018).

8. Hindricks, G. *et al. 2020 ESC Guidelines for the diagnosis and management of atrial fibrillation developed in collaboration with the European Association of Cardio-Thoracic Surgery (EACTS) The Task Force for the diagnosis and management of atrial fibrillation of the Europea*. (2020) doi:10.1093/eurheartj/ehaa612.

9. Marulanda-Londoño, E. & Chaturvedi, S. The Interplay between Obstructive Sleep Apnea and Atrial Fibrillation. *Front Neurol* **8**, 668 (2017).

For peer review only

10. Patil, S. P. *et al.* Treatment of adult obstructive sleep apnea with positive airway pressure: An American academy of sleep medicine systematic review, meta-analysis, and GRADE assessment. *Journal of Clinical Sleep Medicine* vol. 15 301–334 Preprint at https://doi.org/10.5664/jcsm.7638 (2019).

11. McEvoy, R. D. *et al.* CPAP for Prevention of Cardiovascular Events in Obstructive Sleep Apnea. *New England Journal of Medicine* **375**, 919–931 (2016).

12. Yu, J. *et al.* Association of Positive Airway Pressure With Cardiovascular Events and Death in Adults With Sleep Apnea. *JAMA* **318**, 156 (2017).

13. Drager, L. F., McEvoy, R. D., Barbe, F., Lorenzi-Filho, G. & Redline, S. Sleep apnea and cardiovascular disease: Lessons from recent trials and need for team science. *Circulation* **136**, 1840–1850 (2017).

14. Bradley, T. D. & Floras, J. S. Obstructive sleep apnoea and its cardiovascular consequences. *The Lancet* Preprint at https://doi.org/10.1016/S0140-6736(08)61622-0 (2009).

15. Somers, V. K. *et al.* Sleep Apnea and Cardiovascular Disease: An American Heart Association/American College of Cardiology Foundation Scientific Statement From the American Heart Association Council for High Blood Pressure Research Professional Education Committee, Council on . *Circulation* **118**, 1080–1111 (2008).

16. Shen, M. J. & Zipes, D. P. Role of the Autonomic Nervous System in Modulating Cardiac Arrhythmias This Review is in a thematic series on The Autonomic Nervous System and the Cardiovascular System, which includes the following articles: Role of the Autonomic Nervous System in Modula. *Circ Res* **114**, 1004–1021 (2014).

17. Khoo, M. C. K. & Blasi, A. Sleep-related Changes in Autonomic Control in Obstructive Sleep Apnea: A model-based perspective. *Respir Physiol Neurobiol* **188**, 267 (2013).

For peer review only

18. Somers, V. K., Dyken, M. E., Clary, M. P. & Abboud, F. M. Sympathetic neural mechanisms in obstructive sleep apnea. *J Clin Invest* **96**, 1897–904 (1995).

19. Javaheri, S. *et al.* Sleep Apnea: Types, Mechanisms, and Clinical Cardiovascular Consequences. *J Am Coll Cardiol* **69**, 841–858 (2017).

20. Wang, J. *et al.* Impact of Obstructive Sleep Apnea Syndrome on Endothelial Function, Arterial Stiffening, and Serum Inflammatory Markers: An Updated Meta-analysis and Metaregression of 18 Studies. *J Am Heart Assoc* **4**, (2015).

21. Imani, M. M. *et al.* Evaluation of Serum and Plasma Interleukin-6 Levels in Obstructive Sleep Apnea Syndrome: A Meta-Analysis and Meta-Regression. *Front Immunol* **11**, 1343 (2020).

22. Baessler, A. *et al.* Treatment for sleep apnea by continuous positive airway pressure improves levels of inflammatory markers - A meta-analysis. *Journal of Inflammation (United Kingdom)* **10**, 1–10 (2013).

23. Wang, S., Li, S., Wang, B., Liu, J. & Tang, Q. Matrix Metalloproteinase-9 Is a Predictive Factor for Systematic Hypertension and Heart Dysfunction in Patients with Obstructive Sleep Apnea Syndrome. *Biomed Res Int* **2018**, 1569701 (2018).

24. Navarro-García, J. A. *et al.* Fibroblast growth factor-23 promotes rhythm alterations and contractile dysfunction in adult ventricular cardiomyocytes. *Nephrology Dialysis Transplantation* (2019) doi:10.1093/ndt/gfy392.

FIGURE LEGEND

Figure 1. Patient Flow Diagram. *Holter monitor, Blood tests and Questionnaires

For peer review only

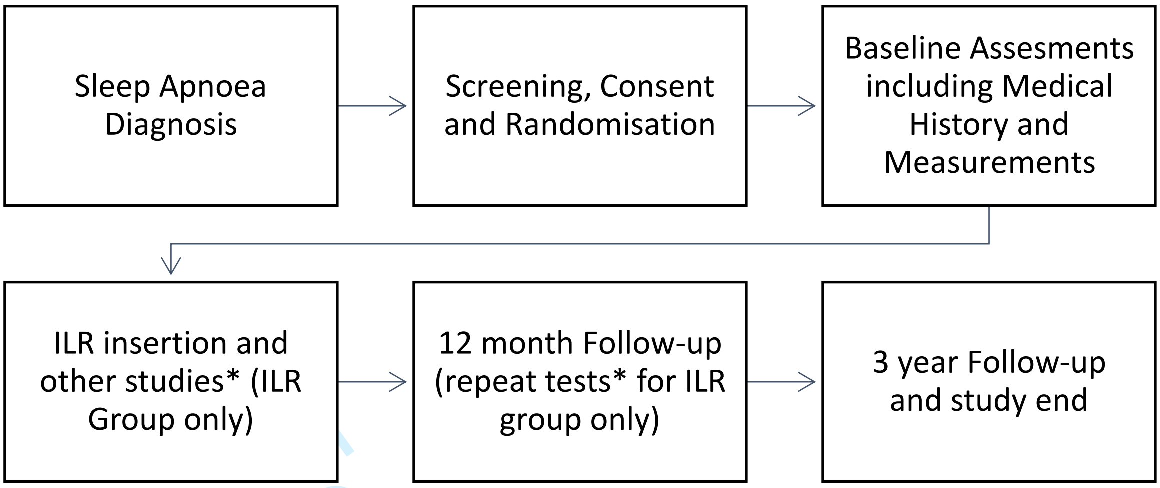

For peer review only

Reporting checklist for protocol of a clinical trial.

Based on the SPIRIT guidelines.

Instructions to authors

Complete this checklist by entering the page numbers from your manuscript where readers will find each of the items listed below.

Your article may not currently address all the items on the checklist. Please modify your text to include the missing information. If you are certain that an item does not apply, please write "n/a" and provide a short explanation.

Upload your completed checklist as an extra file when you submit to a journal.

In your methods section, say that you used the SPIRITreporting guidelines, and cite them as:

Chan A-W, Tetzlaff JM, Gøtzsche PC, Altman DG, Mann H, Berlin J, Dickersin K, Hróbjartsson A, Schulz KF, Parulekar WR, Krleža-Jerić K, Laupacis A, Moher D. SPIRIT 2013 Explanation and Elaboration: Guidance for protocols of clinical trials. BMJ. 2013;346:e7586

		Reporting Item	Page Number
**Administrative information**			
Title	#1	Descriptive title identifying the study design, population, interventions, and, if applicable, trial acronym	1
Trial registration	#2a	Trial identifier and registry name. If not yet registered,	3

For peer review only

		name of intended registry	
Trial registration: data set	#2b	All items from the World Health Organization Trial Registration Data Set	n/a
Protocol version	#3	Date and version identifier	2
Funding	#4	Sources and types of financial, material, and other support	2
Roles and responsibilities: contributorship	#5a	Names, affiliations, and roles of protocol contributors	17
Roles and responsibilities: sponsor contact information	#5b	Name and contact information for the trial sponsor	18
Roles and responsibilities: sponsor and funder	#5c	Role of study sponsor and funders, if any, in study design; collection, management, analysis, and interpretation of data; writing of the report; and the decision to submit the report for publication, including whether they will have ultimate authority over any of these activities	17-18
Roles and responsibilities: committees	#5d	Composition, roles, and responsibilities of the coordinating centre, steering committee, endpoint adjudication committee, data management team, and other individuals or groups overseeing the trial, if applicable (see Item 21a for data monitoring committee)	14

Introduction

Background and rationale	#6a	Description of research question and justification for undertaking the trial, including summary of relevant studies (published and unpublished) examining benefits and harms for each intervention	5
Background and rationale: choice of comparators	#6b	Explanation for choice of comparators	n/a
Objectives	#7	Specific objectives or hypotheses	9
Trial design	#8	Description of trial design including type of trial (eg, parallel group, crossover, factorial, single group), allocation ratio, and framework (eg, superiority, equivalence, non-inferiority, exploratory)	9
**Methods: Participants, interventions, and outcomes**			
Study setting	#9	Description of study settings (eg, community clinic, academic hospital) and list of countries where data will be collected. Reference to where list of study sites can be obtained	9
Eligibility criteria	#10	Inclusion and exclusion criteria for participants. If applicable, eligibility criteria for study centres and individuals who will perform the interventions (eg,	10

For peer review only

		surgeons, psychotherapists)	
Interventions: description	#11a	Interventions for each group with sufficient detail to allow replication, including how and when they will be administered	10
Interventions: modifications	#11b	Criteria for discontinuing or modifying allocated interventions for a given trial participant (eg, drug dose change in response to harms, participant request, or improving / worsening disease)	11
Interventions: adherance	#11c	Strategies to improve adherence to intervention protocols, and any procedures for monitoring adherence (eg, drug tablet return; laboratory tests)	n/a
Interventions: concomitant care	#11d	Relevant concomitant care and interventions that are permitted or prohibited during the trial	n/a
Outcomes	#12	Primary, secondary, and other outcomes, including the specific measurement variable (eg, systolic blood pressure), analysis metric (eg, change from baseline, final value, time to event), method of aggregation (eg, median, proportion), and time point for each outcome. Explanation of the clinical relevance of chosen efficacy and harm outcomes is strongly recommended	9
Participant timeline	#13	Time schedule of enrolment, interventions (including any run-ins and washouts), assessments, and visits for participants. A schematic diagram is highly recommended (see Figure)	12

For peer review only

Sample size	#14	Estimated number of participants needed to achieve study objectives and how it was determined, including clinical and statistical assumptions supporting any sample size calculations	13
Recruitment	#15	Strategies for achieving adequate participant enrolment to reach target sample size	10
**Methods: Assignment of interventions (for controlled trials)**			
Allocation: sequence generation	#16a	Method of generating the allocation sequence (eg, computer-generated random numbers), and list of any factors for stratification. To reduce predictability of a random sequence, details of any planned restriction (eg, blocking) should be provided in a separate document that is unavailable to those who enrol participants or assign interventions	11
Allocation concealment mechanism	#16b	Mechanism of implementing the allocation sequence (eg, central telephone; sequentially numbered, opaque, sealed envelopes), describing any steps to conceal the sequence until interventions are assigned	11
Allocation: implementation	#16c	Who will generate the allocation sequence, who will enrol participants, and who will assign participants to interventions	11

For peer review only

Blinding (masking)	#17a	Who will be blinded after assignment to interventions (eg, trial participants, care providers, outcome assessors, data analysts), and how	n/a
Blinding (masking): emergency unblinding	#17b	If blinded, circumstances under which unblinding is permissible, and procedure for revealing a participant's allocated intervention during the trial	n/a
**Methods: Data collection, management, and analysis**			
Data collection plan	#18a	Plans for assessment and collection of outcome, baseline, and other trial data, including any related processes to promote data quality (eg, duplicate measurements, training of assessors) and a description of study instruments (eg, questionnaires, laboratory tests) along with their reliability and validity, if known. Reference to where data collection forms can be found, if not in the protocol	14
Data collection plan: retention	#18b	Plans to promote participant retention and complete follow-up, including list of any outcome data to be collected for participants who discontinue or deviate from intervention protocols	14
Data management	#19	Plans for data entry, coding, security, and storage, including any related processes to promote data quality (eg, double data entry; range checks for data values).	14

For peer review only

		Reference to where details of data management procedures can be found, if not in the protocol	
Statistics: outcomes	#20a	Statistical methods for analysing primary and secondary outcomes. Reference to where other details of the statistical analysis plan can be found, if not in the protocol	13
Statistics: additional analyses	#20b	Methods for any additional analyses (eg, subgroup and adjusted analyses)	13
Statistics: analysis population and missing data	#20c	Definition of analysis population relating to protocol non-adherence (eg, as randomised analysis), and any statistical methods to handle missing data (eg, multiple imputation)	13
**Methods: Monitoring**			
Data monitoring: formal committee	#21a	Composition of data monitoring committee (DMC); summary of its role and reporting structure; statement of whether it is independent from the sponsor and competing interests; and reference to where further details about its charter can be found, if not in the protocol. Alternatively, an explanation of why a DMC is not needed	n/a
Data monitoring: interim analysis	#21b	Description of any interim analyses and stopping guidelines, including who will have access to these interim results and make the final decision to terminate the trial	n/a
Harms	#22	Plans for collecting, assessing, reporting, and managing solicited and spontaneously reported adverse events and other unintended effects of trial interventions or trial	14

For peer review only

		conduct	
Auditing	#23	Frequency and procedures for auditing trial conduct, if any, and whether the process will be independent from investigators and the sponsor	14
**Ethics and dissemination**			
Research ethics approval	#24	Plans for seeking research ethics committee / institutional review board (REC / IRB) approval	14
Protocol amendments	#25	Plans for communicating important protocol modifications (eg, changes to eligibility criteria, outcomes, analyses) to relevant parties (eg, investigators, REC / IRBs, trial participants, trial registries, journals, regulators)	14
Consent or assent	#26a	Who will obtain informed consent or assent from potential trial participants or authorised surrogates, and how (see Item 32)	14
Consent or assent: ancillary studies	#26b	Additional consent provisions for collection and use of participant data and biological specimens in ancillary studies, if applicable	n/a
Confidentiality	#27	How personal information about potential and enrolled participants will be collected, shared, and maintained in order to protect confidentiality before, during, and after the trial	14
Declaration of	#28	Financial and other competing interests for principal	18

For peer review only

interests		investigators for the overall trial and each study site	
Data access	#29	Statement of who will have access to the final trial dataset, and disclosure of contractual agreements that limit such access for investigators	14
Ancillary and post trial care	#30	Provisions, if any, for ancillary and post-trial care, and for compensation to those who suffer harm from trial participation	11
Dissemination policy: trial results	#31a	Plans for investigators and sponsor to communicate trial results to participants, healthcare professionals, the public, and other relevant groups (eg, via publication, reporting in results databases, or other data sharing arrangements), including any publication restrictions	14
Dissemination policy: authorship	#31b	Authorship eligibility guidelines and any intended use of professional writers	n/a
Dissemination policy: reproducible research	#31c	Plans, if any, for granting public access to the full protocol, participant-level dataset, and statistical code	14
**Appendices**			
Informed consent materials	#32	Model consent form and other related documentation given to participants and authorised surrogates	14
Biological specimens	#33	Plans for collection, laboratory evaluation, and storage of biological specimens for genetic or molecular analysis in the current trial and for future use in ancillary studies, if applicable	n/a

For peer review only

The SPIRIT Explanation and Elaboration paper is distributed under the terms of the Creative Commons Attribution License CC-BY-NC. This checklist was completed on 06. December 2022 using https://www.goodreports.org/, a tool made by the EQUATOR Network in collaboration with Penelope.ai

For peer review only
