# Supplementary material for: Obstructive Sleep Apnoea and Cardiac Arrhythmias (OSCA) trial: a nested cohort study using injectable loop recorders and Holter monitoring in patients with obstructive sleep apnoea
Source: BMJ Open. 2023 Feb 14;13(2):e070884. doi: 10.1136/bmjopen-2022-070884 (PMC9950886; doi:10.1136/bmjopen-2022-070884)
Supplement: Reviewer comments [file bmjopen-2022-070884.reviewer_comments.pdf]

PEER REVIEW HISTORY

BMJ Open publishes all reviews undertaken for accepted manuscripts. Reviewers are asked to complete a checklist review form (**http://bmjopen.bmj.com/site/about/resources/checklist.pdf**) and are provided with free text boxes to elaborate on their assessment. These free text comments are reproduced below.

ARTICLE DETAILS

**TITLE (PROVISIONAL)**	PROTOCOL: Obstructive Sleep Apnoea and Cardiac Arrhythmias (OSCA) Trial – A nested cohort study using Injectable Loop Recorders and Holter monitoring in Obstructive Sleep Apnoea Patients
**AUTHORS**	He, Hejie; Lachlan, Thomas; Chandan, Nakul; Lim, Ven; Kimani, Peter; Ng, G Andre; Ali, Asad; Randeva, Harpal; Osman, Faizel

This article was not externally reviewed at BMJ Open. Protocols that have been independently assessed prior to submission to BMJ Open are usually fast-tracked to publication on the grounds that further substantial changes will not be possible. This independent assessment will usually be external, independent review for both a substantial grant award from a non-commercial or government funder and ethics approval.
